# A Review of Alpha-1 Antitrypsin Binding Partners for Immune Regulation and Potential Therapeutic Application

**DOI:** 10.3390/ijms23052441

**Published:** 2022-02-23

**Authors:** Michael E. O’Brien, Grace Murray, Debananda Gogoi, Azeez Yusuf, Cormac McCarthy, Mark R. Wormald, Michelle Casey, Claudie Gabillard-Lefort, Noel G. McElvaney, Emer P. Reeves

**Affiliations:** 1 Irish Centre for Genetic Lung Disease, Department of Medicine, RCSI University of Medicine and Health Sciences, Beaumont Hospital, D02 YN77 Dublin, Ireland; emmetobrien@rcsi.ie (M.E.O.); gmurray@rcsi.ie (G.M.); debanandagogoi@rcsi.ie (D.G.); azeezyusuf@rcsi.ie (A.Y.); cmccarthy@rcsi.ie (C.M.); michellecasey@rcsi.ie (M.C.); claudiegabillard@rcsi.ie (C.G.-L.); gmcelvaney@rcsi.ie (N.G.M.); 2 Department of Biochemistry, Oxford Glycobiology Institute, University of Oxford, Oxford OX1 3QU, UK; mark.wormald@bioch.ox.ac.uk

**Keywords:** alpha-1 antitrypsin, alpha-1 antitrypsin deficiency, proteases, cytokines, interleukin-6, complement C3, coronavirus disease 2019 (COVID-19)

## Abstract

Alpha-1 antitrypsin (AAT) is the canonical serine protease inhibitor of neutrophil-derived proteases and can modulate innate immune mechanisms through its anti-inflammatory activities mediated by a broad spectrum of protein, cytokine, and cell surface interactions. AAT contains a reactive methionine residue that is critical for its protease-specific binding capacity, whereby AAT entraps the protease on cleavage of its reactive centre loop, neutralises its activity by key changes in its tertiary structure, and permits removal of the AAT-protease complex from the circulation. Recently, however, the immunomodulatory role of AAT has come increasingly to the fore with several prominent studies focused on lipid or protein-protein interactions that are predominantly mediated through electrostatic, glycan, or hydrophobic potential binding sites. The aim of this review was to investigate the spectrum of AAT molecular interactions, with newer studies supporting a potential therapeutic paradigm for AAT augmentation therapy in disorders in which a chronic immune response is strongly linked.

## 1. An Introduction to Alpha-1 Antitrypsin

Alpha-1 antitrypsin is a 52 kDa plasma glycoprotein characterised primarily by its function as an extracellular protease inhibitor of neutrophil elastase (NE). AAT is considered the chief serine protease inhibitor that is encoded by the *SERPINA1* on chromosome 14q32.1-32 [1]. Serine protease inhibitors (SERPINs) share homologous gene regions and have common protein structures [2]. As their name implies, the predominant role of SERPINs is to inhibit their cognate protease, and indeed most SERPINs have evolved in parallel with their specific protease, e.g., antithrombin with thrombin, C1 inhibitor with C1 esterase, and antiplasmin with plasmin [3]. However, the primary function of other extracellular non-inhibitory serine proteases as carrier proteins in plasma is well established, particularly in the case of SERPINA6 (cortisol-binding globulin) and SERPINA7 (thyroxine-binding globulin) [3,4]. The potential role of non-protease AAT–protein interactions has been alluded to in studies of its three-dimensional structure, principally in relation to its corticosteroid-binding domain [5], but also through investigation of candidate binding partners, as summarised in Table 1. However, the status and relative importance of AAT as a carrier protein in the circulation or its non-protease binding at sites of inflammation are incompletely understood at present. Several reports have described electrostatic and hydrophobic protein and peptide interactions with AAT, which highlights the role of AAT beyond protease inhibition [6,7]. In this review, we provide an overview of AAT and the interaction between this antiprotease and its biological environment in order to better understand the function of AAT in health and disease and the emerging therapeutic role of AAT augmentation therapy (alpha 1-proteinase inhibitor) beyond deficiency states. Review of the literature was carried out using the PUBMED database, Google Scholar and The Cochrane Library databases, using several appropriate generic terms.

### Control of Alpha-1 Antitrypsin Production

AAT is abundant in the plasma with a mean concentration of 1.3 g/L (range 0.9–1.75 g/L) and a plasma half-life of 4–5 days. AAT produced by hepatocytes contributes to almost all of the circulating AAT, although it is also produced in smaller quantities by other cells such as monocytes, macrophages, pulmonary alveolar cells and intestinal epithelial cells [31,32,33]. In this regard, *SERPINA1* transcriptional regulation occurs at exons IA, IB and IC, in a tissue-specific manner, as IC regulates transcription in hepatocytes, while IA and IB mediate AAT release in monocytes and macrophages [34,35]. Moreover, *SERPINA1* presents an inflammation-responsive promoter which favours AAT release during inflammation conditions [36,37] and is furthermore reported to be epigenetically regulated by *SERPINA1* promoter methylation [38,39].

**Figure 1:**
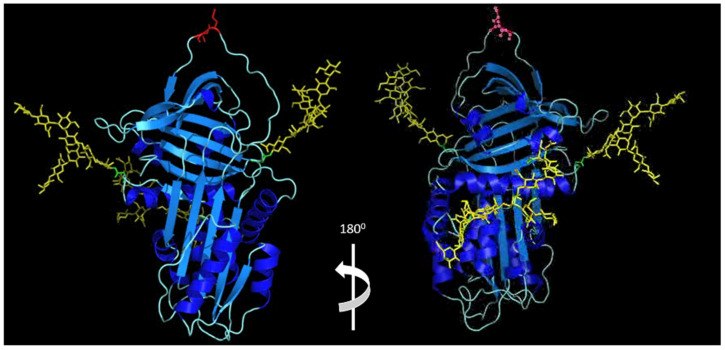
Molecular model of glycosylated alpha-1 antitrypsin. Blue—peptide; yellow—glycans; red—reactive centre loop (peptide linkage) (residues M382-S383). Methods: Molecular modelling was performed on a Silicon Graphics Fuel workstation using InsightII and Discover software (Accelrys Inc., San Diego, USA). Figures were produced using the program Pymol [43]. Protein structures used for modelling were obtained from the pdb database and the structure of glycosylated AAT was based on the crystal structure of human alpha-1 antitrypsin as previously described [44]. The AAT molecule is post-translationally modified by *N*-glycosidically linked oligosaccharides at three asparagine residues at positions 70, 107 and 271.

Following transcription, the tertiary AAT protein structure comprises three β-sheets, nine α-helices and a reactive central loop (RCL) at the C-terminal region [40], a property that is well conserved among other members of the SERPIN superfamily [41] (Figure 1). The reactive methionine residue at position 358 (Met358) is located in the RCL, which extends out from the body of the protein and directs binding to the target protease. From plasma, 80% of AAT diffuses to interstitial tissues, and 0.5–10% reaches biological fluids, including alveolar fluid where concentrations have been measured between 0.1 and 0.3 g/L [42].

AAT has a key role in innate immune defence and during the acute-phase protein response, the plasma concentration of AAT can rise to between two and four-fold under the influence of the pro-inflammatory cytokines interleukin (IL)-6 (IL-6) and IL-1β, and to some extent IL-8, transforming growth factor β (TGF-β) and IL-17 [45]. As a consequence of the acute-phase protein response, increased local and systemic AAT production results in much higher tissue concentrations of AAT, where its ability to bind proteases, proteins, peptides and cytokines, as well as interact with cell surface domains, may have important implications for the regulation of inflammation [18,44]. In turn, cell signalling mechanisms leading to downregulation of AAT production are underexplored, with one in vitro study indicating the ability of AAT itself to downregulate *SERPINA1* mRNA expression in both hepatocytes and peripheral blood mononuclear cells [46].

## 2. Alpha-1 Antitrypsin Deficiency States

**Figure 2:**
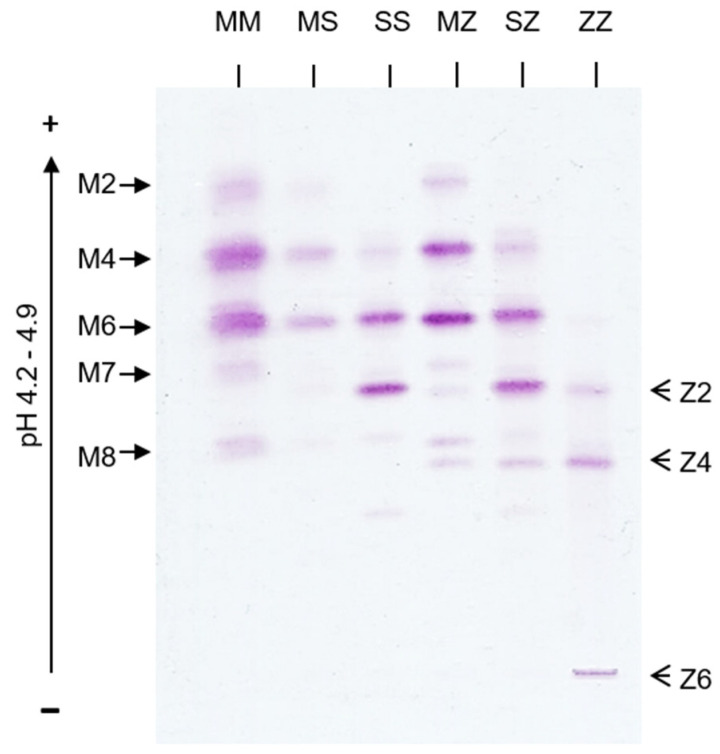
Isoelectric focusing patterns of AAT phenotypes. Healthy control MM AAT glycoforms (M2–M8) are denoted on the left. Glycoforms from an AATD patient homozygous for the Z allele (Z2, Z4 and Z6) are shown on the right.

Much of our understanding of the importance of AAT as an antiprotease, and as an anti-inflammatory molecule, is reliant upon the involvement of patients deficient in AAT in studies exploring its biological effects. Alpha-1 antitrypsin deficiency (AATD) is the best-characterised heritable form of pulmonary emphysema and its discovery was a major breakthrough in our understanding of the role of protease imbalance in pulmonary emphysema [47]. Each *SERPINA1* allele is transmitted by autosomal co-dominant Mendelian inheritance. *SERPINA1* is also polymorphic with over 200 mutations recognised to date that reduce plasma AAT levels by altering protein production and folding, or influencing the glycosylation status of AAT [48]. Several studies identified a variety of COPD-associated mutations/single-nucleotide polymorphisms located at untranslated and promoter regions and introns of *SERPINA1* gene [49,50], which comprise only a small fraction of its disease-associated variants identified so far [51,52]. Mutations are classified by their phenotypic expression and electrophoretic mobility during isoelectric focusing; PiM (medium), PiS (slow), and PiZ (very slow) (Figure 2) [53,54]. The most severe deficiency states are defined by AAT plasma levels less than 35% of the mean expected value (11 μM or 50 mg/dL measured by nephelometry). This is commonly as a result of a point mutation causing an amino acid change from glutamic acid to lysine at position 342 (Glu342Lys), which is referred to as the Z allele. Additionally reported are the PiSZ, PiSS, and rare or null alleles [55].

**Figure 3:**
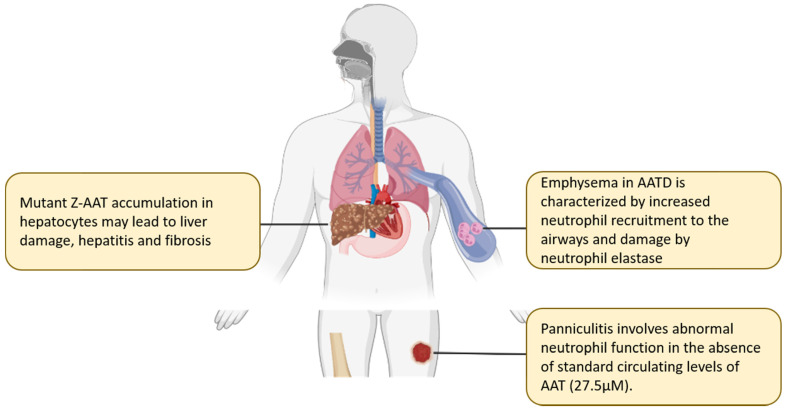
Clinical implications of alpha-1 antitrypsin deficiency. Polymerised aggregates of Z-AAT protein are implicated in the pathogenesis of liver cirrhosis and chronic hepatitis. Accumulation of Z-AAT in hepatocytes leads to impaired secretion of the protein, with individuals homozygous for the Z mutation having 10–15% of normal circulating levels of AAT. Deficiency in AAT results in high influx of neutrophils to the airways, where increased release of serine proteases and uninhibited NE activity can cause damage to lung parenchyma, ultimately leading to emphysema and COPD. In rare cases, AATD is associated with a severe skin condition known as panniculitis and antineutrophil cytoplasmic antibody associated vasculitis (granulomatosis with polyangitis, formally Wegener’s granulomatosis). Panniculitis is characterised by intense neutrophil infiltrates in the subcutaneous tissues and resultant tissue destruction due to the low levels of antiprotease and high levels of protease.

The estimated carrier frequency of the Z allele is 1:25, with a disease incidence of 1:1575 to 1:2100 in some western European populations [56,57]. Homozygous ZZ individuals have a marked reduction in circulating plasma AAT levels to less than 10% of the normal protein concentration. Additionally, the Z-AAT protein is a less competent protease inhibitor than normal healthy type M-AAT and can take twice as long to inhibit a given concentration of NE [58]. In homozygous ZZ individuals, the net effect of reduced circulating AAT protein and diminished antiprotease activity culminates in an ineffective humoral protective shield and a marked protease/antiprotease imbalance, particularly affecting the lung, with the resultant pulmonary disease phenotype arising in these deficiency states (Figure 3). The elastolytic proteases that are released during neutrophil recruitment and activation are the predominant cause for the pathological pulmonary findings in AATD [59], and the resultant burden of disease is the major cause for morbidity and mortality in AATD. Interestingly, there is currently no evidence that AAT levels predict lung disease risk within the SZ cohort. SZ individuals who have never smoked are not at an increased risk of lung disease regardless of their AAT level, while those who currently smoke have a significantly increased risk of airflow obstruction [60].

## 3. Anti-Inflammatory Effects of Alpha-1 Antitrypsin beyond Protease Inhibition

Prior to describing the mechanisms by which AAT can bind inflammatory proteases and mediators, it is important to understand the pleiotropic functions of AAT that mediate a broad range of anti-inflammatory activities beyond protease inhibition [61,62] (Table 2). An example includes the ability of AAT to regulate neutrophil chemotaxis by binding IL-8 and preventing IL-8 interaction with its cognate receptor CXC chemokine receptor 1 (CXCR1) on the neutrophil membrane [7]. It was demonstrated that neutrophils migrate down a functional gradient of AAT in response to an increasing gradient of IL-8, and that glycosylation of AAT is critical for this immunoregulatory effect. Furthermore, AAT prevented immune complex-mediated neutrophil recruitment by modulating disintegrin and metalloprotease domain-17 (ADAM-17) enzymatic activity and shedding of Fc gamma receptor three B (FcγRIIIb) (CD16b) [7]. AAT can also mediate anti-inflammatory effects through the modulation of TNFα signalling. AAT has been shown to bind TNFR thereby preventing TNFα signalling in neutrophils, and to inhibit ADAM-17 activity causing upregulation of TNF receptor 1 (TNF-R1) and reduced TNFα secretion [18]. As a result, AAT promotes an initial augmented response to inflammation in the acute phase followed by selective inhibition later, thereby supporting resolution of chronic inflammation [61]. AAT can also alter neutrophil activity by inducing protein phosphatase 2A (PP2A) activation to prevent the inflammatory and proteolytic responses triggered by TNFα stimulation in the lung [63] and to inhibit TNFα production by monocytes via TLR4 following stimulation with pro-inflammatory cytokines [64].

Illustrative of the effect of the gain-of-function Z mutation, neutrophil apoptosis is accelerated in individuals with AATD by mechanisms involving endoplasmic reticulum (ER) stress and aberrant TNFα signalling [65]. This enhanced neutrophil apoptosis results in decreased neutrophil bactericidal activity, a process that can be ameliorated with AAT augmentation therapy. AAT has also been shown to reduce structural alveolar cell apoptosis independent of elastolytic activity by inhibition of vascular endothelial growth factor (VEGF) receptors with ensuing suppression of caspase-3 activation and oxidative stress [66]. Furthermore, the observation that AAT can inhibit the apoptotic factors, caspase-3 and caspase-1, has widened our perception on the role of AAT in the pathogenesis of emphysema [23,24].

## 4. Alpha-1 Antitrypsin Molecular Interactions

### 4.1. Mechanisms of Binding

A protein’s function is determined by its interaction in the fluid phase (e.g., with components of blood), within the extracellular matrix, at the cell surface, and at target sites such as the pulmonary epithelium or alveolar lining fluid. Some of the recent observations on the anti-inflammatory effects of AAT are independent of its specific antiprotease binding activity. Indeed, proteomic binding studies are uncovering many potential AAT protein interactions, suggesting that AAT may have a myriad of other functional roles other than what has been elucidated to date [16]. Knowledge of the mechanisms through which AAT binds to proteins and peptides beyond protease inhibition permits us to explore the extent of its biological function. The affinity between the surface of a complex protein and potential binding partners within a biological system is often divided between specific and non-specific interactions [75]. Many of the interactions involving AAT that have been described, particularly protease binding, are specific, uniform contacts that result in a molecular structural change that is often irreversible. Non-specific interactions occur across the surface of the molecule and generally do not result in a structural change of either molecule within the macromolecular complex. Instead, they are driven by superimposition of three or four intermolecular interactions (e.g., Van der Waal’s forces, electrostatic, steric, and hydrophobicity) and a multiplicity of structurally dependent weak interactions [76]. Indeed, it is increasingly apparent that AAT has an important role as a carrier protein as supported by its abundance, structural similarities to other lipophilic serine protease carrier proteins, its documented hydrophobic binding domain [5], and the relative specificity for binding hydrophobic proteins compared to other plasma glycoproteins [29]. This is illustrated by observations on AAT binding to LTB4 modulating interaction with cognate receptor BLT1, thereby mediating anti-inflammatory effects through downregulation of immune cell recruitment [6]. Non-specific electrostatic protein interactions observed between AAT and its binding partners are likely dependent on the attached carbohydrate residues or the hydrophilic/hydrophobic surface charge on the AAT protein.

### 4.2. Alpha-1 Antitrypsin-Specific RCL Protease Binding

**Figure 4:**
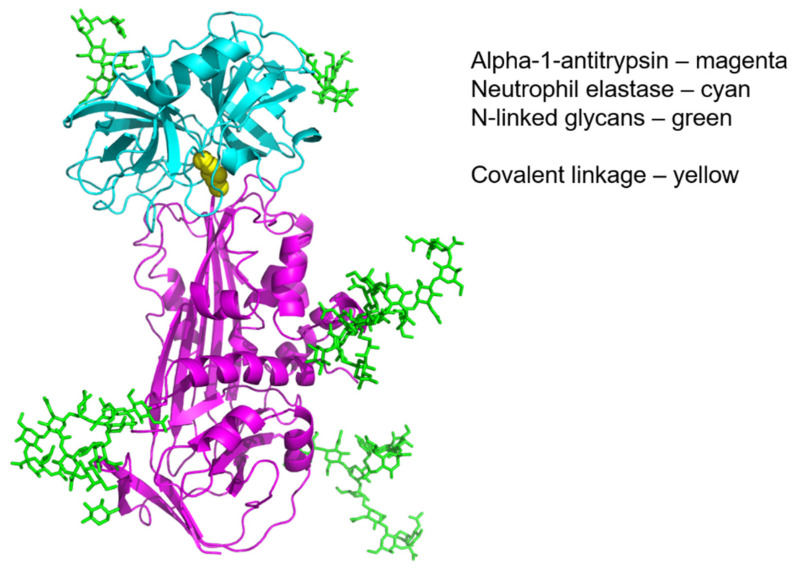
Alpha-1 antitrypsin elastase complex. Model based on the crystal structure of alpha-1 antitrypsin complexed with porcine pancreatic elastase (pdb code 2D26) and the crystal structure of human neutrophil elastase (pdb code 3Q76).

Protease inhibition is central to AAT function and as with all proteins, its structure and function as a serine protease inhibitor are inextricably linked [5]. The primary antiprotease binding activity of AAT has been well characterised, particularly in the case of NE, though it has a wide range of protease inhibitory activity and contributes up to 90% of the total serine protease inhibitory capacity of plasma. The reactivity of the Met358 residue is primarily responsible for the spectrum of protease binding. This amino acid residue has the highest affinity for the serine hydroxyl group on NE to which it binds with an association constant of K = 6.5 × 10^7^ M^−1^ s^−1^, one of the highest binding constants found in nature, and inhibits it in an equimolar ratio (Figure 4). AAT has been shown to inhibit a wide range of other serine proteases including Cathepsin-G (Cath-G) [77], proteinase-3 (PR3) [78], and Factor Xia [79,80] (Table 3). The delay time of inhibition is an important factor regarding the functional effect of AAT in vivo; if it is too long, the protease may have insufficient time to reach its substrate, thus rendering the inhibitor inefficient. In addition, the target enzyme could inactivate the inhibitor by proteolytic attack at a site remote from the active site [77]. In an environment where multiple proteases are active, such as sites of inflammation in vivo, AAT will bind preferentially to NE over other proteases present, e.g., should PR-3 and NE be liberated at the same time and in equal concentrations, 89% of AAT would be bound to NE and 11% bound to PR-3 [81]. Cleavage of the active Met358 by the protease establishes a covalent linkage between the carboxyl group of the serpin reactive site and the serine hydroxyl of the protease. This event triggers a major structural rearrangement that involves loosening of the β-sheets and a kinetically irreversible conformational change by incorporation of the RCL into the β-sheet region of the AAT protein. The translocation of the attached protease by 71 angstrom (Å) from its initial position induces irreversible inactivation of the protease through distortion of the protease active binding site [82]. This mechanism is akin to the function of a mousetrap, with the methionine residue serving as the ‘bait’ that lures the protease to its fateful end [83]. Cleavage of the RCL at Met358 also exposes a new binding pentapeptide domain in the carboxyl terminal fragment of AAT. The inactivated AAT–protease complex is highly stable and can be removed from the circulation through engagement of the newly exposed binding site with the hepatocyte serpin enzyme complex (SEC) receptor [84]. This interaction on the hepatocyte cell surface signals for increased gene expression of *SERPINA1* in a positive feedback loop [85,86].

A point mutation at position 358 can drastically alter the antiprotease function of the AAT molecule by reducing or changing the specificity of this bond for its target protease; this is best illustrated by the rare mutation of Met358 to arginine (AAT-Pittsburgh) resulting in greatly diminished antielastase activity and markedly increased antithrombin activity that results in a fatal bleeding disorder [87]. In addition, the reactive Met358 is a surface exposed methionine residue that is readily oxidised by hydrogen peroxide in cigarette smoke and by oxidising agents released by leukocytes during inflammation [88]. In addition, Met351 and the thiol reactive cysteine-232 (Cys232) residues of AAT are also susceptible to oxidative inactivation [89,90]. Oxidised AAT persists in a functionally inactive form in the circulation, whereby its protease binding capacity is markedly reduced, and fails to stimulate further upregulation of AAT production [91]. However, under certain conditions oxidative inactivation may be physiologically favourable, and necessary for host protease defence, to enable in vivo function of proteases such as NE within a local microenvironment. Interestingly, oxidised AAT has been shown to retain certain anti-inflammatory properties, despite losing its serum elastase inhibitory capacity, as demonstrated through the prevention of neutrophil recruitment to the lungs in a rat model of smoke-induced emphysema [92]. The observed anti-inflammatory mechanism relates to TNFα suppression that provided partial protection to the development of emphysema in this model. Nevertheless, oxidative inactivation of AAT is of major importance in the pathogenesis of emphysematous lung destruction in smokers and it is firmly established that cigarette smoke exposure is the major determinant of an accelerated decline in lung function in AATD causing early death in this population [93]. Additionally, oxidation of the mutant Z-AAT by cigarette smoke can induce Z-AAT polymerisation that may further thwart the residual humoral antiprotease shield (64), which is discussed next [94].

### 4.3. Alpha-1 Antitrypsin RCL Self-Binding Leading to Polymer Formation

A greater understanding of the pathogenesis of AATD was reached on discovery that certain AAT mutants, best described in the case of Z-AAT protein, manifests a gain of function, which causes protein polymerisation or aggregation. In PiZZ homozygotes, the Glu342Lys mutation results in disruption of an intramolecular salt bridge in strand 5 of the five-stranded β-sheet and uncoiling of the upper part of α-helix F [98]. This induces conformational instability of the protein, which involves an initial zero-order conversion of AAT to a polymerogenic monomer intermediate termed M* [99]. Subsequently, a slow concentration-dependent intermolecular association step results in polymerisation through the incorporation of the RCL from an adjacent molecule into the shutter region of the affected β-sheet [58]. Of interest, SERPIN polymerisation and protein aggregation are not unique to AATD; conformational instability of various proteins have been linked to several neurodegenerative processes, including Alzheimer’s disease and Creutzfeldt–Jakob disease [100].

Factors that favour AAT polymerisation in vitro include increased temperature, higher Z-AAT concentration and acidosis, all of which can occur at sites of tissue inflammation in vivo. Consequently, the misfolded protein accumulates within the ER and can be visualised as a ‘beads on a string’ appearance on periodic acid Schiff stain of liver biopsy samples. There is a marked reduction in Z protein egress from the cell leading to ER stress, and thereafter hepatocyte autophagy is overwhelmed and cellular decompensation ensues [101,102]. Due to this mutant gain of function, individuals with severe AATD are at risk of hepatic failure. This is not limited to individuals with the PiZZ phenotype as conformationally unstable AAT variants, such as PiSZ, may also lead to clinically relevant liver disease due to the development of AAT heteropolymers [103]. Of importance, polymerised Z-AAT appear to have inflammatory properties that may contribute to an augmented systemic inflammatory response that influences the clinical phenotype of COPD in AATD [104]. Moreover, AAT polymers may also accumulate in ER of immune cells including monocytes [102] and neutrophils [65], and within bronchial epithelial cells [105]. The accumulation of misfolded Z-AAT in the ER of innate immune cells appears to play a key role in the exaggerated inflammatory response observed in AATD, whereby the accumulation of Z-AAT polymers within the ER of neutrophils leads to ER stress, increased neutrophil apoptosis and defective bacterial killing [65].

A few studies have investigated the potential of autophagy-enhancing drug candidates to treat AATD-mediated liver disease such as phenothiazines, including carbamazepine and fluphenazine, with the aim of degrading mutant Z-AAT that has been retained in the ER of hepatocytes. Both fluphenazine and carbamazepine has been shown to decrease the hepatic load of Z-AAT and hepatic fibrosis in a mouse model of AATD [106,107]. Access to results of clinical trials exploring the impact of therapies aimed at reducing hepatic accumulation of Z-AAT in patients with severe liver disease due to AATD are available online from the publicly available database, https://clinicaltrials.gov/ (accessed on 6 February 2022). Such studies include a phase II clinical trial to determine if carbamazepine therapy leads to a significant reduction in hepatic accumulation of Z-AAT. This latter study was terminated as the number of participants with available pre- and post-treatment biopsies was insufficient to analyse primary and secondary outcomes (NCT01379469) [108]. Clinical trials with small-molecule correctors aimed at correcting misfolding of mutant Z-AAT have been disappointing to date, with phase II clinical trials of VX-814 (NCT04167345) discontinued based on safety and pharmacokinetics data. Furthermore, the results of phase II trials of VX-864 (NCT04474197) resulted in exclusion of the advancement of this molecule into late-stage development. Despite prior failures, investigations are still ongoing into small-molecule correctors and recruitment is currently underway in the UK for a double-blind, randomised, placebo-controlled study assessing the safety and tolerability of the novel compound ZF874, which hopes to act as a molecular ‘patch’ for Z-AAT, allowing the protein to fold correctly and potentially to relieve the hepatocyte burden of polymer accumulation (NCT04443192).

RNA interference (RNAi), known also as post-transcriptional gene silencing, is a natural biological process, whereby short oligonucleotide molecules termed RNAi trigger the silencing of gene expression and thus regulate the expression of protein-coding genes. The objective of potentially employing RNAi therapeutics in AATD therapy would be to cease the production of Z-AAT protein by the liver. This could prevent further accumulation of Z-AAT polymers, halt the progression of liver disease, and enable the gradual clearance of the pre-existing polymers [109]. Clinical trials assessing RNAi candidates, ARC-AAT (NCT02363946), ALN-AAT (NCT02503683), ALN-AAT02 (NCT03767829) as a potential therapeutic for AAT-mediated liver disease were terminated in 2016, 2018 and 2020 based on toxicity concerns in non-human primate studies, low incidence of asymptomatic, transiently elevated liver enzymes in a subset of study subjects and sponsor decision, respectively. This spurred the development of another investigational RNAi therapeutic termed ARO-AAT, which was modified to target hepatocytes through conjugation of *N*-acetylgalactosamine via a linker and therefore did not employ the delivery vehicle (EX1) used in the earlier clinical trial (NCT02363946). Interim results from the AROAAT2002 study (NCT03946449) demonstrated that ARO-AAT was not only well tolerated but also capable of inhibiting Z-AAT expression, reducing intrahepatic Z-AAT accumulation to allow the clearance of Z-AAT polymers and improving liver fibrosis. Phase II trials of AROAAT2001 (SEQUOIA) is underway to evaluate the safety, efficacy and tolerability of multiple doses of the investigational product, ARO-AAT, administered subcutaneously to participants with AATD (NCT03945292) [110]. As of December 2021, the ESTRELLA trial is in the recruitment phase to investigate an alternative RNAi drug named Belcesiran or DCR-A1AT in patients with AATD-associated liver disease (NCT04764448). Thus, there is an exciting number of drugs with potential clinical applications being researched to bridge the gap in therapeutics for the cohort of patients with AATD-mediated liver disease, who currently lack treatment options beyond liver transplantation.

### 4.4. Alpha-1 Antitrypsin Electrostatic Interactions and Post-Translational Glycosylation Effects

The total accessible surface area of AAT (2.34 × 10^4^ Å^2^) is largely hydrophilic in nature, surrounding a hydrophobic core, and all hydrogen bonds are fulfilled on the surface mainly by interactions with main-chain atoms [5]. The surface of AAT has a dipolar characteristic, with the positive pole at the S-359 end and the negative pole at the M-358 end. The isoelectric point (pI) of AAT is 5.37 and therefore it carries a negative charge at physiologic pH. In AATD, the Glu342Lys mutation results in a slight cathodal shift of the isoelectric point by 0.1, resulting in a more negative Z-AAT protein [44,111]. The influence of divalent cations, such as Mg^2+^, Ca^2+^, Cu^2+^, Zn^2+^, and Fe^2+^, is important in modulating AAT protein binding [112] and levels can change during the acute-phase response that may potentially alter the bound protein profile of AAT in inflammatory states [45]. Moreover, AAT undergoes a process of co-translational *N*-glycosylation, resulting in the addition of three oligosaccharide residues contributing 12.5% to the resultant molecular mass of the protein, which may exert electrostatic interactions with potential binding partners.

Comprehensive glycoproteomic analysis of AAT identified glycosylation residues at positions Asn70, Asn107 and Asn271 [113] (Figure 1). *N*-glycosylation takes place initially within the ER, with final glycan branching occurring in the Golgi apparatus. The transfer of oligosaccharides to the selected asparagine residues is catalysed by the enzyme oligosaccharyltransferase, which is present on the luminal surface of the ER membrane [114,115]. This is the central step in *N*-glycosylation. Subsequently, an outer α-1,2-linked glucose residue is trimmed by the enzyme α-glucosidase, followed by the removal of an α-1,3-linked glucose residue by α-glucosidase II, which enables the glycoprotein to interact with soluble and membrane bound lectin chaperones that aid protein folding [116]. Before exiting the ER, a further α-1,3-linked glucose is removed and mannose residues are trimmed by mannosidase I. Within the Golgi, *N*-acetyl-glucosaminyl (GlcNAc) transferase I substitutes GlcNAc residues onto the α-1,3-arm of the high-mannose-type sugar chain, Man5GlcNAc2 [117]. If further glycan branching is possible, this is mediated by GlcNAc transferase II, GlcNAc transferase IV and GlcNAc transferase V forming bi-antennary, tri-antennary and tetra-antennary structures, respectively. Further branch extension by the GlcNac family of enzymes can be inhibited by GlcNAc transferase III. Chain prolongation is often terminated by the addition of a sialic acid residue to a terminal galactose, and this reaction is catalysed by beta-galactoside alpha-2,6-sialyltransferase 1 (ST6GAL1) [118].

Glycosylation of AAT is crucial for its function through prolongation of its plasma half-life, conferring resistance to proteolytic degradation, modulating intermolecular interactions, and the prevention of protein aggregation. To illustrate the relevance and clinical importance of this effect, recombinant non-glycosylated AAT protein produced by bacteria, demonstrates a markedly decreased half-life [119], and is therefore therapeutically ineffective compared to plasma-derived AAT for the purpose of intravenous augmentation therapy [120]. The predominant mechanism for AAT elimination from the body, which is distinct from SEC receptor-mediated protease complex removal, is through the asialoglycoprotein receptor [121]. This is expressed on hepatocytes and on recognition of terminal galactose residues; it expediently removes the protein from the circulation. The addition of sialic acid to terminal glycans shields the residues from receptor binding and thereby prolongs the half-life of AAT [122].

M-AAT glycan expression is modified during the course of community acquired pneumonia (CAP). A glycoform shift arises during the resolving phase of the infection, where there is an increase in circulating levels of sialylated negatively charged AAT glycoforms. This increase in negative AAT glycoforms (termed M0 and M1 AAT) coincides with a decline in the white cell count and C reactive protein levels between days four and six in the course of the infection, and are subsequently cleared by day eight in keeping with clinical recovery. During the resolving phase of CAP, sialylated AAT has a significant binding capacity for positively charged chemokines resulting in inhibition of IL-8-mediated neutrophil chemotaxis, a further confirmation that AAT glycosylation patterns affect protein-protein interactions and modulate immune cell function [17]. More recently, however, a similar glycoform shift has been shown to occur in coronavirus disease 2019 (COVID-19) infection but appears to be associated with worse clinical outcomes [123]. Here, the presence of highly sialylated M0 and M1 glycoforms do not correlate with AAT serum levels or the intensity of the inflammatory response. With focus on AATD, increased core and outer arm fucosylation, including sialyl Lewis-X determinants of the Z-AAT protein, have been characterised [111]. This finding may have implications for the role of Z-AAT as an immunomodulatory protein and its effect upon leukocyte-mediated inflammation in AATD, irrespective of its reduced antiprotease activity. Moreover, a family of at least 32 *SERPINA1* mutations termed null or Q0 have been described [124], which result in the introduction of a premature termination codon in the mRNA coding region [125]. Q0bolton is one of these rare mutations, which results in the production of a truncated 49 kDa Q0bolton-AAT protein, which, despite its altered structure, maintains some antiprotease activity. Seven glycoforms of the Q0bolton-AAT have been identified which demonstrate an altered glycosylation pattern compared to native M-AAT, with an anodal shift and an increased total fucosylation [125]. Q0bolton-AAT possesses increased levels of tri- and tetra-antennary glycans, but lower levels of bi-antennary branching, compared to M-AAT [126]. This trend toward increased core and outer-arm fucosylation differentiates Q0bolton-AAT from M-AAT and is consistent with persistent inflammation [127], although these differences do not appear to impact the binding capacity of Q0bolton-AAT for IL-8.

In summary, glycan residues, and their resultant electrostatic charge, can modulate intermolecular interactions of AAT though binding to the amino acid backbone of proteins (carbohydrate–amino acid interactions). The glycosylation of AAT also protects the protein from glycolysis, prevents protein aggregation, is less polymerogenic, prolongs its plasma half-life, and importantly, supports anti-inflammatory properties whilst not interfering with AAT antiproteinase activity.

### 4.5. Hydrophobic Binding of Alpha-1 Antitrypsin with the Lipoprotein System

Approximately 13% (3.2 × 10^3^ Å^2^) of the accessible surface area of AAT is hydrophobic, with five hydrophobic pockets identified to date. The central hydrophobic core of AAT is filled during relocation of the RCL after protease cleavage or during polymer formation. This site has become a target for drug delivery to prevent loop sheet polymerisation without abolishing the function of AAT [128]. This location is also a potential binding site for other small hydrophobic molecules, such as the potent neutrophil chemoattractant LTB4 [6]. Apolipoprotein B-100 (ApoB100), a major protein component of low-density lipoprotein (LDL) and very low density lipoprotein (VLDL), has previously been identified as a binding partner to AAT [8]. Lipoproteomic analysis of the process of VLDL to LDL conversion has demonstrated that AAT is acquired from plasma or other lipoprotein classes [129]. This may have a particular effect during the acute inflammatory response which is characterised by changes in apolipoprotein synthesis and AAT production [130]. From a pathophysiological perspective, the impact of AAT oxidation was apparent via the formation of AAT–LDL complexes in atherosclerotic plaques that implicates a role for oxidised AAT in atherogenesis. Contrarily, incorporation of AAT into high-density lipoprotein (HDL) may confer some beneficial antielastase properties that protect against atherogenesis [131]. Moreover, it has been reported that enrichment of AAT with HDL afforded better protection against elastase-induced pulmonary emphysema in a murine model than AAT augmentation therapy alone [132].

### 4.6. Alpha-1 Antitrypsin Cysteine Binding Potential

AAT has a single cysteinyl residue (Cys-232) that is situated within a protective crevice due to the close proximity of three lysine residues; this unique structural environment provides the thiolate stabilisation required for a high degree of reactivity across a broad pH range [90]. It has previously been reported that AAT has a strong affinity for monomeric light chain thiolate ions, whereby in vivo complexes between immunoglobulin-κ chains occur without affecting protease inhibitory capacity, which may constitute a mechanism for the linkage and transport of peptides with reactive thiols or disulphides released into plasma and extracellular fluids [11]. Cys-232 is reactive under physiological conditions with proteins and small molecules such as cysteine, glutathione, myeloma immunoglobulin light chains, immunoglobulin A and nitric oxide (NO) [9,10,11]. It has been demonstrated that AAT forms a disulphide bond with the penultimate C-terminal cysteine on the alpha chain of IgA [133], while also retaining its antiprotease activity [134]. AAT can also undergo S-nitrosation, through the interaction of Cys232 with NO formed at the sites of tissue ischaemia or by the action of endothelial or inducible NO synthases at sites of inflammation [30]. The resultant S-NO-AAT molecule is bacteriostatic, can induce vasorelaxation, inhibits platelet aggregation and neutrophil adhesion to endothelial surfaces [30]. AAT thereby may act as a NO reservoir and mediate cytoprotective effects through the attenuation of ischemia-reperfusion injury by maintaining tissue blood flow [135,136]. This property has led to clinical trials in humans utilising AAT augmentation therapy in ST-segment myocardial infarction [137].

### 4.7. The Heparin Binding Motif of Alpha-1 Antitrypsin

The effect of heparin binding to serine proteinase inhibitors is illustrated by the potentiation of antithrombin III activity, a property that is exploited in clinical practice with the use of unfractionated heparin and low-molecular-weight heparin for the purpose of anticoagulation [138]. Binding of heparin is mediated by ionic interactions between its sulphate and carboxylate groups with the positively charged side chains of target proteins. AAT does contain a heparin binding motif, the function of which in the presence of heparin may be to enhance the binding affinity of the reactive Met358 compared to native forms of the AAT protein. However, contrary to this, it has been previously demonstrated that the binding affinity of AAT to NE is reduced in the presence of heparin due to the formation of heparin–elastase complexes; in this study, heparin was not found to bind AAT [139]. Further work is warranted in this area before any definitive conclusion regarding the significance of heparin binding to AAT and its therapeutic potential can be made.

## 5. The Impact of Alpha-1 Antitrypsin Binding in Health and Disease

### 5.1. Alpha-1 Antitrypsin Protease Binding and the Coagulation System

The broad spectrum of AAT protease binding raises the possibility these interactions play a role in the homeostasis of other systems that involve serine protease cleavage, such as the coagulation pathway. Furthermore, this balance may be perturbed in AAT deficiency states. The coagulation cascade is a tightly regulated process and many of the activated coagulation factors are serine proteases. It has been shown previously that AAT accounts for the majority of the plasma inhibition of Factor XIa. However, this has been challenged by the role of other serine protease inhibitors such as C1 inhibitor and α2-antiplasmin through the measurement of Factor XIa–protease inhibitor complexes in blood. Nevertheless, the fact that AAT can bind to fibrinogen in blood [9,10] and is found in significant proportions within formed clot samples [140], indicates that a role for AAT exists not only in the inhibition of clot propagation through control of the coagulation cascade, but also fibrinolysis and the homeostasis of thrombus formation [95]. A reciprocal coupling of coagulation and innate immunity via neutrophil serine proteases has been shown previously [141]. Thrombosis is an important facet of the innate immune response as an additional mechanism to prevent the propagation of microbial invasion [142]. Disseminated intravascular coagulation is a fulminant disease process with a high mortality and previous studies have alluded to the important role that AAT may exert in the humoral response to this devastating complication. It has been postulated that localised fibrin formation may contribute to the pathogenesis of pulmonary emphysema due to increased platelet aggregation potentiating thrombosis leading to a favourable microenvironment for neutrophil attachment [143]. Of particular interest is the role that AAT may play in the protection of fibrinogen from dysregulated proteolysis, particularly by neutrophil-derived proteases [144,145]. A recent study evaluating a specific NE cleavage point in fibrinogen (Aα-Val360) demonstrated that increased fibrinogen cleavage correlated with disease severity in AATD and may be a useful surrogate marker of disease activity in patients with early disease in whom therapeutic intervention may be indicated [146].

### 5.2. Alpha-1 Antitrypsin Protein Complexes and Tissue Inflammation

AAT also has a role in tissue repair and healing through an association with fibronectin. Proteolytically active NE is present in chronic wounds and it was shown that AAT protects fibronectin from enzymatic degradation in wound tissues and is necessary for wound healing [147,148]. There has been significant interest in the role of AAT in the inflammation associated with rheumatoid arthritis, particularly in relation to the formation of IgA–AAT complexes in plasma [149], and in the inflammatory milieu of the synovial fluid of individuals with inflammatory arthritis [150]. This association may relate to the glycosylation status of AAT in plasma during the acute-phase response in inflammatory arthritis [151]. However, there is insufficient evidence to date to indicate that AAT levels correlate with disease activity [152], or indeed that AATD is an independent risk factor for inflammatory arthritis though it may be associated with an increased prevalence of auto-antibody production [153].

### 5.3. Alpha-1 Antitrypsin Binding Partners and the Complement System

Products of complement activation, C3a and C5a, are important neutrophil chemoattractants and complement activation products have been found to be elevated in emphysema [154]. Given the pro-inflammatory properties of C3 activation by-products and the role of AAT in counterbalancing neutrophil-driven inflammation, it is not unexpected that these two abundant plasma proteins may interact. It has been shown that C3b interacts with a range of plasma proteins including AAT, vitamin D binding protein, and α1-acid glycoprotein, by forming high-molecular-weight aggregates through covalent interactions in complement activated serum and plasma [155]. The function of these aggregates is not fully understood at this time and whether they occur in blood in the presence of erythrocytes is not known. Dysregulated complement activation has been described in individuals with AATD, resulting in a diminished capacity to inhibit processing of complement C3 to C3d [16]. Elevated levels of complement fragment C3d have been described in the circulation and airways of patients with AATD, correlating with both the severity of airway obstruction and radiographic pulmonary emphysema. Experiments have shown AAT to bind directly to C3 both in vivo and in vitro, and this is further optimised by AAT glycosylation [16]. Treatment of patients with AATD involves AAT augmentation therapy, which can aid in modulating this uncontrolled complement cascade by disrupting C3 activation and significantly reducing C3d plasma levels when compared with those not on therapy [16]. Moreover, C3d binding to CR3 neutrophil receptors triggered granule release, increased cytokine secretion, and reduced endothelial cell migration and wound healing, with potential implications for AATD-related vasculitis [156].

### 5.4. Alpha-1 Antitrypsin Protease Binding and COVID-19

COVID-19 is a novel emerging infectious disease caused by severe acute respiratory syndrome coronavirus 2 (SARS-CoV-2), first identified in December 2019 in Wuhan, China and classified as a pandemic by the WHO in March 2020. The most serious manifestations of COVID-19 is acute respiratory distress syndrome (ARDS), especially in the older age groups and those with cardiopulmonary disease [157]. Individual variations in susceptibility to and severity of SARS-CoV-2 is likely to be explained by both genetic and non-genetic factors. AATD is just one example of an inheritable condition which may render populations more susceptible to COVID-19. A recent study has shown a significant positive correlation between the combined frequencies of AAT deficiency alleles in 67 countries and their reported COVID-19 mortality rates [158]. The geographical overlap between rates of AATD and severe cases of COVID-19 in Italy has been examined in detail. Genotyping of AAT performed in 3751 Italians from different regions showed a higher prevalence of AATD in northern Italy, the same region that was most affected by SARS-CoV-2 in 2020 [159,160], with 85% of total fatal cases countrywide registered in northern Italy as of 18 April 2020 [161]. These observations suggest that AATD may contribute to regional differences in COVID-19 infection rates, clinical severity and mortality rates, but caution is essential when interpreting these correlations as there are many potential confounding factors. Additional research will be required as the pandemic progresses to further examine this hypothesis and determine the real risk of COVID-19 infection in AATD patients. Nonetheless, the geographical overlap between rates of AATD and severity of COVID-19 suggests that protease–antiprotease imbalance could play a critical role in the pathogenicity and virulence of SARS-CoV-2, or in the host response to COVID-19 infection, and that AAT could be a host protective factor against COVID-19. Other than having a genetic deficiency of AAT, studies have also suggested that during the course of COVID-19 illness, patients may develop an insufficient AAT acute-phase response in severe COVID-19 illness, which may ultimately increase disease severity and risk of mortality [162].

Protease–antiprotease imbalances can arise during the clinical course of COVID-19 infection and recent studies have demonstrated that AAT can bind and inhibit key proteases involved in the pathophysiology of COVID-19, including TMPRSS2 [73,74] and ADAM17 [160] (Table 3). TMPRSS2 is a critical protease in priming of the SARS-CoV-2 spike protein and the host ACE2 receptor prior to viral entrance into the host cell. ADAM17 mediates shedding of ACE2, IL-6 and TNFα, and suppression of ADAM17 may therefore modulate the cytokine storm that has been identified in patients with severe COVID-19. However, evidence to date demonstrates that AAT fails to completely block SARS-CoV-2 entry, possibly due to unprocessed ACE2-mediated cell entry in the absence of TMPRSS2 or the expression of other proteases, which may cleave the S protein [74]. Interestingly, NE, the prime target of AAT, has been proposed to act as an alternative spike priming protease [163]. AAT has been shown to suppress SARS-CoV-2 viral replication in cell lines and primary cells including human airway epithelial cultures [73,74]. Taken together, these findings suggest AAT may play a critical role in the innate immune defence against SARS-CoV-2 infection and highlight AAT as a potential drug candidate in the treatment of COVID-19 [162]. Moreover, increased sialylation of AAT in COVID-19 is documented in the literature. AAT immunophenotyping performed on 25 COVID-19 patients in ICU demonstrated an AAT glycoform shift, which appears to be associated with worse clinical outcomes [123]. Highly sialylated M0 and M1 AAT glycoforms were identified in all those who died and in 59% of patients who survived the illness. The synthesis of more negatively charged glycoforms correlated with a higher NE inhibitory capacity ratio, but not with AAT serum levels or the intensity of the inflammatory response. The study postulates that the qualitative shift in AAT glycoforms is an attempt to trigger antielastase activity and boost the anti-inflammatory response [123], as has been reported in patients with community-acquired pneumonia [17], but unfortunately this appears futile as this modification appears to correlate with negative outcomes in COVID-19.

## 6. AAT Augmentation Therapy

### AAT Replacement Therapy in Acute and Chronic Disease

Efforts to restore normal circulating plasma levels of AAT in AATD individuals culminated in the development of AAT augmentation therapy in the 1980s from pooled donor plasma [164]. Initial studies demonstrated safe and effective delivery of the purified AAT protein to maintain levels above a putative protective threshold of 0.5 g/dL (11 μmol/L) and augmentation therapy was approved in the United States by the Food and Drug Administration (FDA) based on biochemical efficacy [165]. The first randomised controlled trial of intravenous plasma purified AAT was performed recently, which demonstrated slowing of emphysema progression when measured by computer tomography determined lung density [166]. Weekly treatment doses of AAT higher than the FDA-approved standard dose (60 mg/kg/week) are not currently recommended [167]. Results of a recent pilot study, however, have demonstrated that double dose AAT therapy (120 mg/kg/week) is not only well tolerated but may provide additional clinical benefits. Double dosing was found to be effective at further reducing the level of serine proteases in both the airway and circulation, reducing elastin degradation, and diminishing airway inflammation when compared to standard dose therapy [168,169]. The RAPID Programme also demonstrated that biweekly dosing with 120 mg/kg of AAT is a safe, well tolerated and a convenient alternative to the dosing regimen currently recommended by the FDA [170]. The SPARTA trial is currently ongoing in a number of European countries to further explore the efficacy and safety of AAT in subjects with pulmonary emphysema due to AATD (NCT01983241). Moreover, the use of nebulised AAT overcomes some of the shortcomings of intravenous therapy and permits delivery to the local site of inflammation [171] and the ability of recombinant AAT to neutralise NE is preserved using this approach [172]. However, recombinant AAT has not been shown to modulate markers of inflammation; such an effect has only been observed using plasma purified glycosylated AAT to date [173].

The alternative biological effects of AAT, specifically its potential anti-inflammatory and antiapoptotic properties, have led to the speculative use of AAT augmentation therapy in a range of conditions. In this regard, the beneficial effect of AAT was observed in ischaemia–reperfusion injury after myocardial infarction [25]. Subsequently, the first clinical trial outside of AATD was conducted using single-dose augmentation therapy in acute ST elevation myocardial infarction [137]. In this study, augmentation therapy was found to be safe and well tolerated with some blunting of the acute inflammatory response.

A growing body of evidence from preclinical studies has demonstrated that AAT may have therapeutic potential in autoimmune diseases. AAT activity is altered in both developing and established type I diabetes mellitus, as well as in established type II diabetes [174]. Promising results from murine models of pancreatic allograft transplantation [175,176] have culminated in clinical trials for onset type I diabetes (NCT02093221 and NCT01183468) [137,177,178]. AAT supplementation was found to be well tolerated and safe; however, its clinical benefit in type I diabetes remains inconclusive. A higher dose of AAT (>90 mg/kg/week) may be needed for optimal therapeutic effect [179,180]. Systemic lupus erythematous (SLE) is an autoimmune disorder in which reactive dendritic cells appear to play a critical role in disease development and pathogenesis. A mouse model of lupus has demonstrated that AAT can inhibit the activation and functioning of dendritic cells, and can attenuate autoimmunity and renal damage [181]. A more recent study identified that treatment with AAT can prevent lupus development and extend the lifespan of lupus prone mice [182].

Current evidence suggests that loss of AAT in salivary gland cells with a consequent increase in elastase expression could contribute to the initiation of primary Sjogren’s syndrome [183], although the efficacy of AAT replacement therapy in this condition has not been assessed. A recent clinical trial has found AAT infusions to be well tolerated and demonstrated potential efficacy in the treatment of steroid-refractory severe acute graft-versus host disease [184,185], but additional studies are warranted and further clinical trials remain ongoing (NCT03805789, NCT04167514).

Reports and trials indicated the AAT have significant role in COVID-19 infection (NCT04799873; NCT04495101) [161,186]. Finally, there are currently several clinical trials evaluating the therapeutic potential of AAT in hospitalised patients with COVID-19 worldwide, including USA, Brazil and Chile (NCT04547140), Saudi Arabia (NCT04385836) and Ireland (EudraCT 2020-001391-15) [187]. The latter trial explored administration of IV plasma-purified AAT on circulating plasma levels of IL-6 in COVID-19 patients who required invasive and non-invasive respiratory support [187]. In turn, the first successful administration of IV AAT for severe COVID-19 complicated by ARDS was reported in a patient with cystic fibrosis [188]. Systemic and airway inflammatory markers, particularly IL-6, IL-1β, IL-8 and NE, were elevated in the patient’s samples prior to AAT administration. A clinical improvement was observed two days after AAT administration, which was accompanied by a decrease in inflammation. Promising results from a clinical trial in Germany (NCT04799873) investigating the effect of both inhaled and combined inhaled/IV AAT administration on the clinical course of nine patients with mild to moderate COVID-19 have been published, with all patients treated with AAT surviving and displaying an eventual improvement in respiratory function before hospital discharge [189].

## 7. Conclusions

Alpha-1 antitrypsin (AAT) is the canonical serine protease inhibitor that has been the subject of extensive study. Deficiency of AAT is associated with a heritable form of pulmonary emphysema that is characterised by a markedly reduced humoral protease inhibitory shield, in particular against the effects of neutrophil-derived proteases. AAT can inhibit a broad array of other proteases to varying degrees, which may mediate important biological effects on account of its abundance in plasma. Increasingly, it is recognised that AAT has diverse interactions beyond protease inhibition that have been shown to facilitate beneficial anti-inflammatory and antiapoptotic responses. Uncovering the protease and novel non-protease binding properties of AAT has led to a deeper understanding of the function of this protein in health and disease. Knowledge of the full interaction profile of AAT as it circulates in health and in deficiency states may lead to a deeper understanding of its effects, uncover novel mechanisms of action, and ultimately lead to innovative therapeutic applications of augmentation therapy in a variety of disease states.

## Figures and Tables

**Table 1:** Known binding partners to alpha-1 antitrypsin in health and disease.

	Binding Partner	Disease Processes	Reference
**Plasma and** **circulating cells**	Apolipoprotein B-100	Atheroma	[8]
	IgA complexes	Rheumatoid arthritis, myeloma	[9]
	Fibrinogen	Healthy	[10]
	IgK light chains	Myeloma	[11]
	HSP 70	Diabetes mellitus	[12]
	Grp94	Diabetes mellitus	[13]
	PSA/kallikrein 3	Benin prostatic hypertrophy, prostate cancer	[14]
	Cholesterol	Not specified	[15]
	Leukotriene B4	Inflammation	[6]
	Complement C3	Complement activation	[16]
	Complement C4-A	Not specified	[16]
	Serum albumin	Not specified	[16]
	Apolipoprotein A-I	Not specified	[16]
	Prothrombin	Not specified	[16]
	IL-8	Emphysema/pneumonia	[7,17]
	TNFR	Emphysema	[18]
	FcγRIIIb (CD16b)	Biomarker of pulmonary exacerbation	[19]
**Intracellular**	Heme	Not specified	[20]
	Prohepcidin	Iron metabolism	[21]
	Retinoic acid	Emphysema	[22]
	Caspase 3	Apoptosis, emphysema	[23]
	Caspase 1	Apoptosis	[24,25]
	Calpain 1	Neutrophil activation	[26]
**Extracellular/Tissue**	IgA complexes	Synovial fluid/rheumatoid arthritis	[9,10]
	Surfactant protein A	Airway surface liquid	[27]
	Aggrecanase 1	Synovial tissue/OA	[28]
	Bile acids	Bile	[29]
	NO	Inflammation/innate immunity	[30]

Previously published binding partners to AAT are categorised by biological compartment. The involvement of the respective protein and disease process is provided where applicable. Glossary: IgA = immunoglobulin A. BPH = benign prostatic hypertrophy. OA = osteoarthritis.

**Table 2:** Pleiotropic functions of alpha-1 antitrypsin.

Role of Alpha-1 Antitrypsin	Function	Reference
Protease inhibitor	Anti-NE, -Cath-G and -PR3	[47]
Anti-apoptosis	Inhibition of caspase-1, caspase-3, and calpain-1	[23,25,26]
Antioxidant	Oxidative stress inhibition	[67]
Anti-inflammatory/tissue repair	Repair, fibroblast proliferation, procollagen synthesis, and activation of MAP kinase pathways	[68]
	Modulation of ADAM-17 activity	[28]
	Substrate for metalloproteinase MMP-9 activity	[69]
	Inactivation of matriptase in vitro and inhibition of epithelial sodium transport in vitro and in vivo	[70]
Antibacterial	Bacteriostasis—binding to furin (inhibits bacterial toxin activation)	[71]
Antiviral	Inhibition of HIV-1 viral cell entry	[72]
	Inhibition of SARS-CoV-2 entry by inhibiting transmembrane serine protease 2 and ADAM-17	[73,74]

**Table 3:** Known serine proteases that bind and are inhibited by alpha-1 antitrypsin.

Proteinase	AAT	Oxidised AAT	Reference
Neutrophil Elastase	6.5 ± 4.0 × 10^7^	3.1 ± 0.2 × 10^4^	[77]
Proteinase 3	8.1 × 10^6^	-	[78]
Cathepsin G	4.1 ± 0.6 × 10^5^	6.5 ± 0.3 × 10^2^	[77]
Chymotrypsin	5.4 ± 0.6 × 10^6^	1.0 ± 0.4 × 10^6^	[77]
Trypsin 2 (Anionic)	7.3 ± 1.8 × 10^4^	3.2 ± 0.1 × 10^4^	[77]
Trypsin 1 (Cationic)	1.1 ± 0.2 × 10^4^	3.0 ± 1.1 × 10^3^	[77]
Factor Xia	1.3 × 10^4^	-	[80]
Matriptase	3.1 × 10^2^	0	[70]
Plasmin	1.9 ± 0.1 × 10^2^	0	[95]
Thrombin	4.8 ± 0.5 × 10^1^	0	[96]
Activated Protein C	1.1 × 10^1^	-	[97]
Transmembrane Serine Protease 2	-	-	[74]

## References

[1] Schroeder W.T., Miller M.F., Woo S.L., Saunders G.F. (1985). Chromosomal localization of the human alpha 1-antitrypsin gene (PI) to 14q31–32. Am. J. Hum. Genet..

[2] Hunt L.T., Dayhoff M.O. (1980). A surprising new protein superfamily containing ovalbumin, antithrombin-III, and alpha1-proteinase inhibitor. Biochem. Biophys. Res. Commun..

[3] Van Gent D., Sharp P., Morgan K., Kalsheker N. (2003). Serpins: Structure, function and molecular evolution. Int. J. Biochem. Cell Biol..

[4] Hammond G.L., Smith C.L., Paterson N.A.M., Sibbald W.J. (1990). A role for corticosteroid-binding globulin in delivery of cortisol to activated neutrophils. J. Clin. Endocrinol. Metab..

[5] Huber R., Carrell R.W. (1989). Implications of the three-dimensional structure of alpha.1-antitrypsin for structure and function of serpins. Biochemistry.

[6] O’Dwyer C.A., O’Brien M.E., Wormald M.R., White M.M., Banville N., Hurley K., McCarthy C., McElvaney N.G., Reeves E.P. (2015). The BLT1 inhibitory function of α-1 antitrypsin augmentation therapy disrupts leukotriene B4 neutrophil signaling. J. Immunol..

[7] Bergin D.A., Reeves E.P., Meleady P., Henry M., McElvaney O.J., Carroll T.P., Condron C., Chotirmall S.H., Clynes M., O’Neill S.J. (2010). α-1 antitrypsin regulates human neutrophil chemotaxis induced by soluble immune complexes and IL-8. J. Clin. Investig..

[8] Mashiba S., Wada Y., Takeya M., Sugiyama A., Hamakubo T., Nakamura A., Noguchi N., Niki E., Izumi A., Kobayashi M. (2001). In Vivo complex formation of oxidized α1-antitrypsin and LDL. Arter. Thromb. Vasc. Biol..

[9] Tomasi T.B., Hauptman S.P. (1974). The binding of alpha-1 antitrypsin to human IgA. J. Immunol..

[10] Laurell C.B., Thulin E. (1975). Complexes in human plasma between α1-antitrypsin and IgA, and α1-antitrypsin and fibrinogen. Scand. J. Immunol..

[11] Laurell C.B., Thulin E. (1974). Complexes in plasma between light chain κ immunoglobulins and α1-antitrypsin respectively prealbumin. Immunochemistry.

[12] Finotti P., Pagetta A. (2004). A heat shock protein70 fusion protein with α1-antitrypsin in plasma of Type 1 diabetic subjects. Biochem. Biophys. Res. Commun..

[13] Pagetta A., Folda A., Brunati A.M., Finotti P. (2003). Identification and purification from the plasma of Type 1 diabetic subjects of a proteolytically active Grp94: Evidence that Grp94 is entirely responsible for plasma proteolytic activity. Diabetologia.

[14] Zhang W.-M., Finne P., Leinonen J., Vesalainen S., Nordling S., Stenman U.-H. (1999). Measurement of the complex between prostate-specific antigen and α1-protease inhibitor in serum. Clin. Chem..

[15] Janciauskiene S., Eriksson S. (1993). In vitro complex formation between cholesterol and α1-proteinase inhibitor. FEBS Lett..

[16] O’Brien M.E., Fee L., Browne N., Carroll T.P., Meleady P., Henry M., McQuillan K., Murphy M.P., Logan M., McCarthy C. (2020). Activation of complement component 3 is associated with airways disease and pulmonary emphysema in alpha-1 antitrypsin deficiency. Thorax.

[17] McCarthy C., Dunlea D.M., Saldova R., Henry M., Meleady P., McElvaney O.J., Marsh B., Rudd P.M., Reeves E.P., McElvaney N.G. (2018). Glycosylation repurposes alpha-1 antitrypsin for resolution of community-acquired pneumonia. Am. J. Respir. Crit. Care Med..

[18] Bergin D.A., Reeves E.P., Hurley K., Wolfe R., Jameel R., Fitzgerald S., McElvaney N.G. (2014). The circulating proteinase inhibitor α-1 antitrypsin regulates neutrophil degranulation and autoimmunity. Sci. Transl. Med..

[19] Reeves E.P., Bergin D.A., Fitzgerald S., Hayes E., Keenan J., Henry M., Meleady P., Vega-Carrascal I., Murray M.A., Low T.B. (2012). A novel neutrophil derived inflammatory biomarker of pulmonary exacerbation in cystic fibrosis. J. Cyst. Fibros..

[20] Karnaukhova E., Krupnikova S.S., Rajabi M., Alayash A.I. (2012). Heme binding to human alpha-1 proteinase inhibitor. Biochim. Biophys. Acta Gen. Subj..

[21] Pandur E., Nagy J., Poór V.S., Sarnyai Á., Huszár A., Miseta A., Sipos K. (2009). α-1 antitrypsin binds preprohepcidin intracellularly and prohepcidin in the serum. FEBS J..

[22] Karnaukhova E. (2010). Interactions of α1–proteinase inhibitor with small ligands of therapeutic potential: Binding with retinoic acid. Amino Acids.

[23] Petrache I., Fijalkowska I., Medler T.R., Skirball J., Cruz P., Zhen L., Petrache H.I., Flotte T.R., Tuder R.M. (2006). Alpha-1 antitrypsin inhibits caspase-3 activity, preventing lung endothelial cell apoptosis. Am. J. Pathol..

[24] Wang Y., He Y., Abraham B., Rouhani F.N., Brantly M.L., Scott D.E., Reed J.L. (2012). Cytosolic, autocrine alpha-1 proteinase inhibitor (A1PI) inhibits caspase-1 and blocks IL-1β dependent cytokine release in monocytes. PLoS ONE.

[25] Toldo S., Seropian I.M., Mezzaroma E., Van Tassell B.W., Salloum F.N., Lewis E.C., Voelkel N., Dinarello C.A., Abbate A. (2011). Alpha-1 antitrypsin inhibits caspase-1 and protects from acute myocardial ischemia–reperfusion injury. J. Mol. Cell. Cardiol..

[26] Al-Omari M., Korenbaum E., Ballmaier M., Lehmann U., Jonigk D., Manstein D.J., Welte T., Mahadeva R., Janciauskiene S. (2011). Acute-phase protein α1-antitrypsin inhibits neutrophil calpain I and induces random migration. Mol. Med..

[27] Gorrini M., Lupi A., Iadarola P., Dos Santos C., Rognoni P., Dalzoppo D., Carrabino N., Pozzi E., Baritussio A., Luisetti M. (2005). SP-A binds alpha1-antitrypsin in vitro and reduces the association rate constant for neutrophil elastase. Respir. Res..

[28] Yoshida K., Suzuki Y., Saito A., Fukuda K., Hamanishi C., Munakata H. (2005). Aggrecanase-1 (ADAMTS-4) interacts with α1-antitrypsin. Biochim. Biophys. Acta Gen. Subj..

[29] Janciauskiene S., Eriksson S. (1994). The interaction of hydrophobic bile acids with the α1-proteinase inhibitor. FEBS Lett..

[30] Miyamoto Y., Akaike T., Maeda H. (2000). S-Nitrosylated human α1-protease inhibitor. Biochim. Biophys. Acta Protein Struct. Mol. Enzym..

[31] Perlmutter D.H., Cole F.S., Kilbridge P., Rossing T.H., Colten H.R. (1985). Expression of the alpha 1-proteinase inhibitor gene in human monocytes and macrophages. Proc. Natl. Acad. Sci. USA.

[32] Mulgrew A.T., Taggart C., Lawless M.W., Greene C., Brantly M.L., O’Neill S.J., McElvaney N.G. (2004). Z α1-antitrypsin polymerizes in the lung and acts as a neutrophil chemoattractant. Chest.

[33] Molmenti E.P., Perlmutter D.H., Rubin D.C. (1993). Cell-specific expression of alpha 1-antitrypsin in human intestinal epithelium. J. Clin. Investig..

[34] Perlino E., Cortese R., Ciliberto G. (1987). The human alpha 1-antitrypsin gene is transcribed from two different promoters in macrophages and hepatocytes. EMBO J..

[35] Kalsheker N., Morley S., Morgan K. (2002). Gene regulation of the serine proteinase inhibitors alpha1-antitrypsin and alpha1-antichymotrypsin. Biochem. Soc. Trans..

[36] Yuan Y., Diciaccio B., Li Y., Elshikha A.S., Titov D., Brenner B., Seifer L., Pan H., Karic N., Akbar M.A. (2018). Anti-inflammaging effects of human alpha-1 antitrypsin. Aging Cell.

[37] Knoell D.L., Ralston D.R., Coulter K.R., Wewers M.D. (1998). Alpha 1-antitrypsin and protease complexation is induced by lipopolysaccharide, interleukin-1β, and tumor necrosis factor-α in monocytes. Am. J. Respir. Crit. Care Med..

[38] Rotondo J.C., Oton-Gonzalez L., Selvatici R., Rizzo P., Pavasini R., Campo G.C., Lanzillotti C., Mazziotta C., De Mattei M., Tognon M. (2020). *SERPINA1* gene promoter is differentially methylated in peripheral blood mononuclear cells of pregnant women. Front. Cell Dev. Biol..

[39] Beckmeyer-Borowko A., Imboden M., Rezwan F.I., Wielscher M., Amaral A.F.S., Jeong A., Schaffner E., Auvinen J., Sebert S., Karhunen V. (2018). *SERPINA1* methylation and lung function in tobacco-smoke exposed European children and adults: A meta-analysis of ALEC population-based cohorts. Respir. Res..

[40] Loebermann H., Tokuoka R., Deisenhofer J., Huber R. (1984). Human α1-proteinase inhibitor: Crystal structure analysis of two crystal modifications, molecular model and preliminary analysis of the implications for function. J. Mol. Biol..

[41] Ryu S.-E., Choi H.-J., Kwon K.-S., Lee K.N., Yu M.-H. (1996). The native strains in the hydrophobic core and flexible reactive loop of a serine protease inhibitor: Crystal structure of an uncleaved α1-antitrypsin at 2.7 Å. Structure.

[42] Ogushi F., Fells G.A., Hubbard R.C., Straus S.D., Crystal R.G. (1987). Z-type alpha 1-antitrypsin is less competent than M1-type alpha 1-antitrypsin as an inhibitor of neutrophil elastase. J. Clin. Investig..

[43] Schrodinger L. (2010). The PyMOL Molecular Graphics System.

[44] McCarthy C., Saldova R., Wormald M.R., Rudd P.M., McElvaney N.G., Reeves E.P. (2014). The role and importance of glycosylation of acute phase proteins with focus on alpha-1 antitrypsin in acute and chronic inflammatory conditions. J. Proteome Res..

[45] Gabay C., Kushner I. (1999). Acute-phase proteins and other systemic responses to inflammation. N. Engl. J. Med..

[46] Karadagi A., Johansson H., Zemack H., Salipalli S., Mörk L.-M., Kannisto K., Jorns C., Gramignoli R., Strom S., Stokkeland K. (2017). Exogenous alpha 1-antitrypsin down-regulates *SERPINA1* expression. PLoS ONE.

[47] Gadek J.E., Fells G.A., Zimmerman R.L., Rennard S.I., Crystal R.G. (1981). Antielastases of the human alveolar structures. Implications for the protease-antiprotease theory of emphysema. J. Clin. Investig..

[48] Ferrarotti I., Thun G.A., Zorzetto M., Ottaviani S., Imboden M., Schindler C., Von Eckardstein A., Rohrer L., Rochat T., Russi E.W. (2012). Serum levels and genotype distribution of α1-antitrypsin in the general population. Thorax.

[49] Corley M., Solem A., Phillips G., Lackey L., Ziehr B., Vincent H.A., Mustoe A.M., Ramos S.B.V., Weeks K.M., Moorman N.J. (2017). An RNA structure-mediated, posttranscriptional model of human α-1-antitrypsin expression. Proc. Natl. Acad. Sci. USA.

[50] Chappell S., Daly L., Morgan K., Guetta Baranes T., Roca J., Rabinovich R., Millar A., Donnelly S.C., Keatings V., MacNee W. (2005). Cryptic haplotypes of SERPINA 1 confer susceptibility to chronic obstructive pulmonary disease. Hum. Mutat..

[51] American Thoracic Society, European Respiratory Society (2003). American Thoracic Society/European Respiratory Society statement: Standards for the diagnosis and management of individuals with alpha-1 antitrypsin deficiency. Am. J. Respir. Crit. Care Med..

[52] Abboud R.T., Nelson T.N., Jung B., Mattman A. (2011). Alpha1-antitrypsin deficiency: A clinical-genetic overview. Appl. Clin. Genet..

[53] Pannell R., Johnson D., Travis J. (1974). Isolation and properties of human plasma α-1-proteinase inhibitor. Biochemistry.

[54] Fagerhol M.K., Laurell C.-B. (1967). The polymorphism of “prealbumins” and α1-antitrypsin in human sera. Clin. Chim. Acta.

[55] Greene C.M., Marciniak S.J., Teckman J., Ferrarotti I., Brantly M.L., Lomas D.A., Stoller J.K., McElvaney N.G. (2016). α1-antitrypsin deficiency. Nat. Rev. Dis. Prim..

[56] Sveger T. (1976). Liver disease in alpha1-antitrypsin deficiency detected by screening of 200,000 infants. N. Engl. J. Med..

[57] Carroll T.P., O’Connor C.A., Floyd O., McPartlin J., Kelleher D.P., O’Brien G., Dimitrov B.D., Morris V.B., Taggart C.C., McElvaney N.G. (2011). The prevalence of alpha-1 antitrypsin deficiency in Ireland. Respir. Res..

[58] Lomas D.A., Evans D.L., Stone S.R., Chang W.S.W., Carrell R.W. (1993). Effect of the Z mutation on the physical and inhibitory properties of alpha1-antitrypsin. Biochemistry.

[59] Taggart C.C., Greene C.M., Carroll T.P., O’Neill S.J., McElvaney N.G. (2005). Elastolytic proteases: Inflammation resolution and dysregulation in chronic infective lung disease. Am. J. Respir. Crit. Care Med..

[60] Franciosi A.N., Hobbs B.D., McElvaney O.J., Molloy K., Hersh C., Clarke L., Gunaratnam C., Silverman E.K., Carroll T.P., McElvaney N.G. (2020). Clarifying the risk of lung disease in SZ alpha-1 antitrypsin deficiency. Am. J. Respir. Crit. Care Med..

[61] Lockett A.D., Kimani S., Ddungu G., Wrenger S., Tuder R.M., Janciauskiene S.M., Petrache I. (2013). α1-antitrypsin modulates lung endothelial cell inflammatory responses to TNF-α. Am. J. Respir. Cell Mol. Biol..

[62] Lewis E.C. (2012). Expanding the clinical indications for α1-antitrypsin therapy. Mol. Med..

[63] Geraghty P., Eden E., Pillai M., Campos M., McElvaney N.G., Foronjy R.F. (2014). α1-antitrypsin activates protein phosphatase 2A to counter lung inflammatory responses. Am. J. Respir. Crit. Care Med..

[64] Nita I.M., Serapinas D., Janciauskiene S.M. (2007). α1-antitrypsin regulates CD14 expression and soluble CD14 levels in human monocytes in vitro. Int. J. Biochem. Cell Biol..

[65] Hurley K., Lacey N., O’Dwyer C.A., Bergin D.A., McElvaney O.J., O’Brien M.E., McElvaney O.F., Reeves E.P., McElvaney N.G. (2014). Alpha-1 antitrypsin augmentation therapy corrects accelerated neutrophil apoptosis in deficient individuals. J. Immunol..

[66] Petrache I., Fijalkowska I., Zhen L., Medler T.R., Brown E., Cruz P., Choe K.-H., Taraseviciene-Stewart L., Scerbavicius R., Shapiro L. (2006). A novel antiapoptotic role for α1-antitrypsin in the prevention of pulmonary emphysema. Am. J. Respir. Crit. Care Med..

[67] Janciauskiene S. (2020). The beneficial effects of antioxidants in health and diseases. Chronic Obstr. Pulm. Dis. J. COPD Found..

[68] Dabbagh K., Laurent G.J., Shock A., Leoni P., Papakrivopoulou J., Chambers R.C. (2001). Alpha-1-antitrypsin stimulates fibroblast proliferation and procollagen production and activates classical MAP kinase signalling pathways. J. Cell. Physiol..

[69] Liu Z., Zhou X., Shapiro S.D., Shipley J.M., Twining S.S., Diaz L.A., Senior R.M., Werb Z. (2000). The serpin α1-proteinase inhibitor is a critical substrate for gelatinase B/MMP-9 in vivo. Cell.

[70] Janciauskiene S., Nita I., Subramaniyam D., Li Q., Lancaster J.R., Matalon S. (2008). α1-antitrypsin inhibits the activity of the matriptase catalytic domain in vitro. Am. J. Respir. Cell Mol. Biol..

[71] Hada K., Isshiki K., Matsuda S., Yuasa K., Tsuji A. (2013). Engineering of alpha1-antitrypsin variants with improved specificity for the proprotein convertase furin using site-directed random mutagenesis. Protein Eng. Des. Sel..

[72] Münch J., Ständker L., Adermann K., Schulz A., Schindler M., Chinnadurai R., Pöhlmann S., Chaipan C., Biet T., Peters T. (2007). Discovery and optimization of a natural HIV-1 entry inhibitor targeting the gp41 fusion peptide. Cell.

[73] Wettstein L., Weil T., Conzelmann C., Müller J.A., Groß R., Hirschenberger M., Seidel A., Klute S., Zech F., Prelli Bozzo C. (2021). Alpha-1 antitrypsin inhibits TMPRSS2 protease activity and SARS-CoV-2 infection. Nat. Commun..

[74] Azouz N.P., Klingler A.M., Callahan V., Akhrymuk I.V., Elez K., Raich L., Henry B.M., Benoit J.L., Benoit S.W., Noé F. (2021). Alpha 1 antitrypsin is an inhibitor of the SARS-CoV-2–priming protease TMPRSS2. Pathog. Immun..

[75] Jones S., Thornton J.M. (1996). Principles of protein-protein interactions. Proc. Natl. Acad. Sci. USA.

[76] Wang W., Woodbury N.W. (2014). Selective protein–peptide interactions at surfaces. Acta Biomater..

[77] Beatty K., Bieth J., Travis J. (1980). Kinetics of association of serine proteinases with native and oxidized alpha-1-proteinase inhibitor and alpha-1-antichymotrypsin. J. Biol. Chem..

[78] Duranton J., Bieth J.G. (2003). Inhibition of proteinase 3 by α1-antitrypsin in vitro predicts very fast inhibition in vivo. Am. J. Respir. Cell Mol. Biol..

[79] Wuillemin W.A., Minnema M., Meijers J.C., Roem D., Eerenberg A.J., Nuijens J.H., ten Cate H., Hack C.E. (1995). Inactivation of factor XIa in human plasma assessed by measuring factor XIa-protease inhibitor complexes: Major role for C1-inhibitor. Blood.

[80] Scott C.F., Schapira M., James H.L., Cohen A.B., Colman R.W. (1982). Inactivation of factor XIa by plasma protease inhibitors: Predominant role of alpha 1-protease inhibitor and protective effect of high molecular weight kininogen. J. Clin. Investig..

[81] Rao N.V., Wehner N.G., Marshall B.C., Gray W.R., Gray B.H., Hoidal J.R. (1991). Characterization of proteinase-3 (PR-3), a neutrophil serine proteinase. Structural and functional properties. J. Biol. Chem..

[82] Huntington J.A., Read R.J., Carrell R.W. (2000). Structure of a serpin–protease complex shows inhibition by deformation. Nature.

[83] Lomas D.A., Parfrey H. (2004). α1-antitrypsin deficiency •4: Molecular pathophysiology. Thorax.

[84] Perlmutter D.H., Joslin G., Nelson P., Schasteen C., Adams S.P., Fallon R.J. (1990). Endocytosis and degradation of alpha 1-antitrypsin-protease complexes is mediated by the serpin-enzyme complex (SEC) receptor. J. Biol. Chem..

[85] Joslin G., Wittwer A., Adams S., Tollefsen D.M., August A., Perlmutter D.H. (1993). Cross-competition for binding of alpha 1-antitrypsin (alpha 1 AT)-elastase complexes to the serpin-enzyme complex receptor by other serpin-enzyme complexes and by proteolytically modified alpha 1 AT. J. Biol. Chem..

[86] Joslin G., Fallon R.J., Bullock J., Adams S.P., Perlmutter D.H. (1991). The SEC receptor recognizes a pentapeptide neodomain of alpha 1-antitrypsin-protease complexes. J. Biol. Chem..

[87] Owen M.C., Brennan S.O., Lewis J.H., Carrell R.W. (1983). Mutation of antitrypsin to antithrombin. Alpha 1-antitrypsin Pittsburgh (358 Met leads to Arg), a fatal bleeding disorder. N. Engl. J. Med..

[88] Carp H., Miller F., Hoidal J.R., Janoff A. (1982). Potential mechanism of emphysema: Alpha 1-proteinase inhibitor recovered from lungs of cigarette smokers contains oxidized methionine and has decreased elastase inhibitory capacity. Proc. Natl. Acad. Sci. USA.

[89] Taggart C., Cervantes-Laurean D., Kim G., McElvaney N.G., Wehr N., Moss J., Levine R.L. (2000). Oxidation of either methionine 351 or methionine 358 in alpha 1-antitrypsin causes loss of anti-neutrophil elastase activity. J. Biol. Chem..

[90] Griffiths S.W., King J., Cooney C.L. (2002). The reactivity and oxidation pathway of cysteine 232 in recombinant human α1-antitrypsin. J. Biol. Chem..

[91] Yu S.-D., Gan J.C. (1977). The role of sialic acid and galactose residues in determining the survival of human plasma α1-antitrypsin in the blood circulation. Arch. Biochem. Biophys..

[92] Churg A., Wang R.D., Xie C., Wright J.L. (2003). α-1-antitrypsin ameliorates cigarette smoke–induced emphysema in the mouse. Am. J. Respir. Crit. Care Med..

[93] O’Brien M.E., Pennycooke K., Carroll T., Shum J., Fee L.T., O’Connor C., Logan P.M., Reeves E.P., McElvaney N.G. (2015). The impact of smoke exposure on the clinical phenotype of alpha-1 antitrypsin deficiency in Ireland: Exploiting a National Registry to understand a rare disease. COPD J. Chronic Obstr. Pulm. Dis..

[94] Alam S., Li Z., Janciauskiene S., Mahadeva R. (2011). Oxidation of Z α1-antitrypsin by cigarette smoke induces polymerization: A novel mechanism of early-onset emphysema. Am. J. Respir. Cell Mol. Biol..

[95] Levi M., Roem D., Kamp A.M., De Boer J.P., Hack C.E., Cate J.W.T. (1993). Assessment of the relative contribution of different protease inhibitors to the inhibition of plasmin in vivo. Thromb. Haemost..

[96] Fredenburgh J.C., Stafford A.R., Weitz J.I. (2001). Conformational changes in thrombin when complexed by serpins. J. Biol. Chem..

[97] Heeb M.J., Bischoff R., Courtney M., Griffin J. (1990). Inhibition of activated protein C by recombinant alpha 1-antitrypsin variants with substitution of arginine or leucine for methionine358. J. Biol. Chem..

[98] Gooptu B., Hazes B., Chang W.-S.W., Dafforn T.R., Carrell R.W., Read R.J., Lomas D.A. (2000). Inactive conformation of the serpin alpha 1-antichymotrypsin indicates two-stage insertion of the reactive loop: Implications for inhibitory function and conformational disease. Proc. Natl. Acad. Sci. USA.

[99] Yamasaki M., Li W., Johnson D.J.D., Huntington J.A. (2008). Crystal structure of a stable dimer reveals the molecular basis of serpin polymerization. Nature.

[100] Carrell R.W., Lomas D.A. (2002). Alpha1-antitrypsin deficiency—A model for conformational diseases. N. Engl. J. Med..

[101] Lawless M.W., Greene C.M., Mulgrew A., Taggart C.C., O’Neill S.J., McElvaney N.G. (2004). Activation of endoplasmic reticulum-specific stress responses associated with the conformational disease Z α1-antitrypsin deficiency. J. Immunol..

[102] Carroll T.P., Greene C.M., O’Connor C.A., Nolan A.M., O’Neill S.J., McElvaney N.G. (2010). Evidence for unfolded protein response activation in monocytes from individuals with α-1 antitrypsin deficiency. J. Immunol..

[103] Mahadeva R., Chang W.-S.W., Dafforn T.R., Oakley D.J., Foreman R.C., Calvin J., Wight D.G., Lomas D.A. (1999). Heteropolymerization of S, I, and Z α1-antitrypsin and liver cirrhosis. J. Clin. Investig..

[104] Mahadeva R., Atkinson C., Li Z., Stewart S., Janciauskiene S., Kelley D.G., Parmar J., Pitman R., Shapiro S.D., Lomas D.A. (2005). Polymers of Z α1-antitrypsin co-localize with neutrophils in emphysematous alveoli and are chemotactic in vivo. Am. J. Pathol..

[105] Pini L., Tiberio L., Venkatesan N., Bezzi M., Corda L., Luisetti M., Ferrarotti I., Malerba M., Lomas D.A., Janciauskiene S. (2014). The role of bronchial epithelial cells in the pathogenesis of COPD in Z-alpha-1 antitrypsin deficiency. Respir. Res..

[106] Li J., Pak S.C., O’Reilly L.P., Benson J.A., Wang Y., Hidvegi T., Hale P., Dippold C., Ewing M., Silverman G.A. (2014). Fluphenazine reduces proteotoxicity in *C. elegans* and mammalian models of alpha-1-antitrypsin deficiency. PLoS ONE.

[107] Hidvegi T., Ewing M., Hale P., Dippold C., Beckett C., Kemp C., Maurice N., Mukherjee A., Goldbach C., Watkins S. (2010). An autophagy-enhancing drug promotes degradation of mutant α1-antitrypsin Z and reduces hepatic fibrosis. Science.

[108] Washington University School of Medicine (2021). Carbamazepine in Severe Liver Disease Due to Alpha-1 Antitrypsin Deficiency (CBZ). https://clinicaltrials.gov/ct2/show/results/NCT01379469.

[109] Wooddell C.I., Blomenkamp K., Peterson R.M., Subbotin V.M., Schwabe C., Hamilton J., Chu Q., Christianson D.R., Hegge J.O., Kolbe J. (2020). Development of an RNAi therapeutic for alpha-1-antitrypsin liver disease. JCI Insight.

[110] Santos G., Turner A.M. (2020). Alpha-1 antitrypsin deficiency: An update on clinical aspects of diagnosis and management. Fac. Rev..

[111] McCarthy C., Saldova R., O’Brien M.E., Bergin D.A., Carroll T.P., Keenan J., Meleady P., Henry M., Clynes M., Rudd P.M. (2014). Increased outer arm and core fucose residues on the N-glycans of mutated alpha-1 antitrypsin protein from alpha-1 antitrypsin deficient individuals. J. Proteome Res..

[112] Dudev T., Lim C. (2003). Principles governing Mg, Ca, and Zn binding and selectivity in proteins. Chem. Rev..

[113] Kolarich D., Weber A., Turecek P.L., Schwarz H.-P., Altmann F. (2006). Comprehensive glyco-proteomic analysis of human α1-antitrypsin and its charge isoforms. Proteomics.

[114] Rudd P.M., Dwek R.A. (1997). Glycosylation: Heterogeneity and the 3D structure of proteins. Crit. Rev. Biochem. Mol. Biol..

[115] Kelleher D.J., Gilmore R. (2006). An evolving view of the eukaryotic oligosaccharyltransferase. Glycobiology.

[116] Trombetta E.S., Helenius A. (1998). Lectins as chaperones in glycoprotein folding. Curr. Opin. Struct. Biol..

[117] Schachter H. (1986). Biosynthetic controls that determine the branching and microheterogeneity of protein-bound oligosaccharides. Biochem. Cell Biol..

[118] Kuhn B., Benz J., Greif M., Engel A.M., Sobek H., Rudolph M.G. (2013). The structure of human α-2,6-sialyltransferase reveals the binding mode of complex glycans. Acta Crystallogr. Sect. D Biol. Crystallogr..

[119] Casolaro M.A., Fells G., Wewers M., Pierce J.E., Ogushi F., Hubbard R., Sellers S., Forstrom J., Lyons D., Kawasaki G. (1987). Augmentation of lung antineutrophil elastase capacity with recombinant human alpha-1-antitrypsin. J. Appl. Physiol..

[120] Karnaukhova E., Ophir Y., Golding B. (2006). Recombinant human alpha-1 proteinase inhibitor: Towards therapeutic use. Amino Acids.

[121] Mast A.E., Enghild J.J., Pizzo S.V., Salvesen G. (1991). Analysis of the plasma elimination kinetics and conformational stabilities of native, proteinase-complexed and reactive site cleaved serpins: Comparison of alpha-1-proteinase inhibitor, alpha-1-antichymotrypsin, antithrombin III, alpha-2-antiplasmin, angiotensinogen, and ovalbumin. Biochemistry.

[122] Lusch A., Kaup M., Marx U., Tauber R., Blanchard V., Berger M. (2013). Development and analysis of alpha 1-antitrypsin neoglycoproteins: The impact of additional N-glycosylation sites on serum half-life. Mol. Pharm..

[123] Zerimech F., Jourdain M., Onraed B., Bouchecareilh M., Sendid B., Duhamel A., Balduyck M., Pigny P. (2021). LICORNE Study Groupa Protease-antiprotease imbalance in patients with severe COVID-19. Clin. Chem. Lab. Med..

[124] Ferrarotti I., Carroll T.P., Ottaviani S., Fra A.M., O’Brien G., Molloy K., Corda L., Medicina D., Curran D.R., McElvaney N.G. (2014). Identification and characterisation of eight novel *SERPINA1* Null mutations. Orphanet J. Rare Dis..

[125] Reeves E.P., O’Dwyer C.A., Dunlea D.M., Wormald M.R., Hawkins P., Alfares M., Kotton D.N., Rowe S.M., Wilson A.A., McElvaney N.G. (2018). Ataluren, a new therapeutic for alpha-1 antitrypsin–deficient individuals with nonsense mutations. Am. J. Respir. Crit. Care Med..

[126] Reeves E.P., Dunlea D.M., McQuillan K., O’Dwyer C.A., Carroll T.P., Saldova R., Akepati P.R., Wormald M.R., McElvaney O.J., Shutchaidat V. (2019). Circulating truncated alpha-1 antitrypsin glycoprotein in patient plasma retains anti-inflammatory capacity. J. Immunol..

[127] Goodarzi M.T., Turner G.A. (1998). Reproducible and sensitive determination of charged oligosaccharides from haptoglobin by PNGase F digestion and HPAEC/PAD analysis: Glycan composition varies with disease. Glycoconj. J..

[128] Patschull A.O.M., Gooptu B., Ashford P., Daviter T., Nobeli I. (2012). In silico assessment of potential druggable pockets on the surface of α1-antitrypsin conformers. PLoS ONE.

[129] Sun H.-Y., Chen S.-F., Lai M.-D., Chang T.-T., Chen T.-L., Li P.-Y., Shieh D.-B., Young K.-C. (2010). Comparative proteomic profiling of plasma very-low-density and low-density lipoproteins. Clin. Chim. Acta.

[130] Kumaraswamy S.B., Linder A., Akesson P., Dahlback B. (2012). Decreased plasma concentrations of apolipoprotein M in sepsis and systemic inflammatory response syndromes. Crit. Care.

[131] Ortiz-Muñoz G., Houard X., Martín-Ventura J.-L., Ishida B.Y., Loyau S., Rossignol P., Moreno J.-A., Kane J.P., Chalkley R.J., Burlingame A.L. (2009). HDL antielastase activity prevents smooth muscle cell anoikis, a potential new antiatherogenic property. FASEB J..

[132] Moreno J.-A., Ortega-Gomez A., Rubio-Navarro A., Louedec L., Ho-Tin-Noé B., Caligiuri G., Nicoletti A., Levoye A., Plantier L., Meilhac O. (2014). High-density lipoproteins potentiate α1-antitrypsin therapy in elastase-induced pulmonary emphysema. Am. J. Respir. Cell Mol. Biol..

[133] Vaerman J.P., Hagiwara K., Kobayashi K., Rits M. (1987). Complexes of albumin and α1-antitrypsin with Fc-fragment of IgA monomer are disulfide-bound to penultimate C-terminal cysteine in the Cα3-domain. Immunol. Lett..

[134] Musiani P., Lauriola L., Piantelli M. (1978). Inhibitory activity of alpha-1-antitrypsin bound to human IgA. Clin. Chim. Acta.

[135] Moldthan H.L., Hirko A.C., Thinschmidt J.S., Grant M.B., Li Z., Peris J., Lu Y., Elshikha A.S., King M.A., Hughes J.A. (2014). Alpha 1-antitrypsin therapy mitigated ischemic stroke damage in rats. J. Stroke Cerebrovasc. Dis..

[136] Gao W., Zhao J., Kim H., Xu S., Chen M., Bai X., Toba H., Cho H.-R., Zhang H., Keshavjeel S. (2014). α1-antitrypsin inhibits ischemia reperfusion-induced lung injury by reducing inflammatory response and cell death. J. Hear. Lung Transplant..

[137] Abbate A., Van Tassell B.W., Christopher S., Abouzaki N.A., Sonnino C., Oddi C., Carbone S., Melchior R.D., Gambill M.L., Roberts C.S. (2015). Effects of prolastin C (plasma-derived alpha-1 antitrypsin) on the acute inflammatory response in patients with ST-segment elevation myocardial infarction (from the VCU-alpha 1-RT pilot study). Am. J. Cardiol..

[138] Rosenberg R.D., Damus P.S. (1973). The purification and mechanism of action of human antithrombin-heparin cofactor. J. Biol. Chem..

[139] Frommherz K.J., Faller B., Bieth J.G. (1991). Heparin strongly decreases the rate of inhibition of neutrophil elastase by alpha 1-proteinase inhibitor. J. Biol. Chem..

[140] Talens S., Malfliet J.J.M.C., van Hal P.T.W., Leebeek F.W.G., Rijken D.C. (2013). Identification and characterization of α1-antitrypsin in fibrin clots. J. Thromb. Haemost..

[141] Massberg S., Grahl L., von Bruehl M.-L., Manukyan D., Pfeiler S., Goosmann C., Brinkmann V., Lorenz M., Bidzhekov K., Khandagale A.B. (2010). Reciprocal coupling of coagulation and innate immunity via neutrophil serine proteases. Nat. Med..

[142] Opal S.M. (2000). Phylogenetic and functional relationships between coagulation and the innate immune response. Crit. Care Med..

[143] Diamond M.S., Springer T.A. (1993). A subpopulation of Mac-1 (CD11b/CD18) molecules mediates neutrophil adhesion to ICAM-1 and fibrinogen. J. Cell Biol..

[144] Weitz J.I., Huang A.J., Landman S.L., Nicholson S.C., Silverstein S.C. (1987). Elastase-mediated fibrinogenolysis by chemoattractant-stimulated neutrophils occurs in the presence of physiologic concentrations of antiproteinases. J. Exp. Med..

[145] Carter R., Mumford R.A., Treonze K.M., Finke P.E., Davies P., Si Q., Humes J.L., Dirksen A., Piitulainen E., Ahmad A. (2011). The fibrinogen cleavage product alpha-Val360, a specific marker of neutrophil elastase activity in vivo. Thorax.

[146] Carter R.I., Ungurs M.J., Pillai A., Mumford R.A., Stockley R.A. (2015). The relationship of the fibrinogen cleavage biomarker Aa-Val360 with disease severity and activity in α1-antitrypsin deficiency. Chest.

[147] Rao C.N., Ladin D.A., Liu Y.Y., Chilukuri K., Hou Z.Z., Woodley D.T. (1995). α1-antitrypsin is degraded and non-functional in chronic wounds but intact and functional in acute wounds: The inhibitor protects fibronectin from degradation by chronic wound fluid enzymes. J. Investig. Dermatol..

[148] Grinnell F., Zhu M. (1996). Fibronectin degradation in chronic wounds depends on the relative levels of elastase, α1-proteinase inhibitor, and α2-macroglobulin. J. Investig. Dermatol..

[149] Scott L.J., Evans E.L., Dawes P.T., Russell G.I., Mattey D.L. (1998). Comparison of IgA-alpha1-antitrypsin levels in rheumatoid arthritis and seronegative oligoarthritis: Complex formation is not associated with inflammation per se. Br. J. Rheumatol..

[150] Swedlund H.A., Hunder G.G., Gleich G.J. (1974). Alpha 1-antitrypsin in serum and synovial fluid in rheumatoid arthritis. Ann. Rheum. Dis..

[151] Chrostek L., Cylwik B., Gińdzieńska-Sieśkiewicz E., Gruszewska E., Szmitkowski M., Sierakowski S. (2014). Sialic acid level reflects the disturbances of glycosylation and acute-phase reaction in rheumatic diseases. Rheumatol. Int..

[152] Cylwik B., Chrostek L., Gindzienska-Sieskiewicz E., Sierakowski S., Szmitkowski M. (2010). Relationship between serum acute-phase proteins and high disease activity in patients with rheumatoid arthritis. Adv. Med. Sci..

[153] McCarthy C., Orr C., Fee L.T., Carroll T., Dunlea D.M., Hunt D., Dunne E., O’Connell P., McCarthy G., Kenny D. (2017). Brief report: Genetic variation of the α1-antitrypsin gene is associated with increased autoantibody production in rheumatoid arthritis. Arthritis Rheumatol..

[154] Marc M.M., Korosec P., Kosnik M., Kern I., Flezar M., Suskovic S., Sorli J. (2004). Complement factors C3a, C4a, and C5a in chronic obstructive pulmonary disease and asthma. Am. J. Respir. Cell Mol. Biol..

[155] Ramadass M., Ghebrehiwet B., Smith R.J., Kew R.R. (2013). Generation of multiple fluid-phase C3b: Plasma protein complexes during complement activation: Possible implications in C3 glomerulopathies. J. Immunol..

[156] Fee L.T., Gogoi D., O’Brien M.E., McHugh E., Casey M., Gough C., Murphy M., Hopkins A.M., Carroll T.P., McElvaney N.G. (2021). C3d elicits neutrophil degranulation and decreases endothelial cell migration, with implications for patients with alpha-1 antitrypsin deficiency. Biomedicines.

[157] Zhou F., Yu T., Du R., Fan G., Liu Y., Liu Z., Xiang J., Wang Y., Song B., Gu X. (2020). Clinical course and risk factors for mortality of adult inpatients with COVID-19 in Wuhan, China: A retrospective cohort study. Lancet.

[158] Shapira G., Shomron N., Gurwitz D. (2020). Ethnic differences in alpha--1 antitrypsin deficiency allele frequencies may partially explain national differences in COVID--19 fatality rates. FASEB J..

[159] Massi G., Cotumaccio R., Auconi P. (1982). Alpha-1-antitrypsin (α1AT) phenotypes and PiM subtypes in Italy. Evidence of considerable geographic variability. Hum. Genet..

[160] De Loyola M.B., dos Reis T.T.A., de Oliveira G.X.L.M., Palmeira J., Argañaraz G.A., Argañaraz E.R. (2021). Alpha--1--antitrypsin: A possible host protective factor against COVID--19. Rev. Med. Virol..

[161] Vianello A., Braccioni F. (2020). Geographical overlap between alpha-1 antitrypsin deficiency and COVID-19 infection in Italy: Casual or causal?. Arch. Bronconeumol..

[162] McElvaney O.J., McEvoy N.L., McElvaney O.F., Carroll T.P., Murphy M.P., Dunlea D.M., Ni Choileain O., Clarke J., O’Connor E., Hogan G. (2020). Characterization of the inflammatory response to severe COVID-19 illness. Am. J. Respir. Crit. Care Med..

[163] Bhattacharyya C., Das C., Ghosh A., Singh A.K., Mukherjee S., Majumder P.P., Basu A., Biswas N.K. (2021). SARS-CoV-2 mutation 614G creates an elastase cleavage site enhancing its spread in high AAT-deficient regions. Infect. Genet. Evol..

[164] Wewers M.D., Casolaro M.A., Sellers S.E., Swayze S.C., McPhaul K.M., Wittes J.T., Crystal R.G. (1987). Replacement therapy for alpha 1-antitrypsin deficiency associated with emphysema. N. Engl. J. Med..

[165] Crystal R.G. (1990). Alpha 1-antitrypsin deficiency, emphysema, and liver disease. Genetic basis and strategies for therapy. J. Clin. Investig..

[166] Chapman K.R., Burdon J.G.W., Piitulainen E., Sandhaus R.A., Seersholm N., Stocks J.M., Stoel B.C., Huang L., Yao Z., Edelman J.M. (2015). Intravenous augmentation treatment and lung density in severe α1 antitrypsin deficiency (RAPID): A randomised, double-blind, placebo-controlled trial. Lancet.

[167] Sandhaus R.A., Turino G., Brantly M.L., Campos M., Cross C.E., Goodman K., Hogarth D.K., Knight S.L., Stocks J.M., Stoller J.K. (2016). The diagnosis and management of alpha-1 antitrypsin deficiency in the adult. Chronic Obstr. Pulm. Dis. J. COPD Found..

[168] Campos M.A., Kueppers F., Stocks J.M., Strange C., Chen J., Griffin R., Wang-Smith L., Brantly M.L. (2013). Safety and pharmacokinetics of 120 mg/kg versus 60 mg/kg weekly intravenous infusions of alpha-1 proteinase inhibitor in alpha-1 antitrypsin deficiency: A multicenter, randomized, double-blind, crossover study (SPARK). COPD J. Chronic Obstr. Pulm. Dis..

[169] Campos M.A., Geraghty P., Holt G., Mendes E., Newby P.R., Ma S., Luna-Diaz L.V., Turino G.M., Stockley R.A. (2019). The biological effects of double-dose alpha-1 antitrypsin augmentation therapy. A pilot clinical trial. Am. J. Respir. Crit. Care Med..

[170] Greulich T., Chlumsky J., Wencker M., Vit O., Fries M., Chung T., Shebl A., Vogelmeier C., Chapman K.R., McElvaney N.G. (2018). Safety of biweekly α1-antitrypsin treatment in the RAPID programme. Eur. Respir. J..

[171] McElvaney N.G., Hubbard R.C., Birrer P., Chernick M.S., Caplan D.B., Frank M.M., Crystal R.G. (1991). Aerosol α1-antitrypsin treatment for cystic fibrosis. Lancet.

[172] Martin S.L., Downey D., Bilton D., Keogan M.T., Edgar J., Elborn J.S. (2006). Safety and efficacy of recombinant alpha1-antitrypsin therapy in cystic fibrosis. Pediatr. Pulmonol..

[173] Griese M., Latzin P., Kappler M., Weckerle K., Heinzlmaier T., Bernhardt T., Hartl D. (2006). Alpha1-antitrypsin inhalation reduces airway inflammation in cystic fibrosis patients. Eur. Respir. J..

[174] Park S.S., Rodriguez Ortega R., Agudelo C.W., Perez J.P., Gandara B.P., Garcia-Arcos I., McCarthy C., Geraghty P. (2021). Therapeutic potential of alpha-1 antitrypsin in Type 1 and Type 2 diabetes mellitus. Medicina.

[175] Lewis E.C., Shapiro L., Bowers O.J., Dinarello C.A. (2005). Alpha1-antitrypsin monotherapy prolongs islet allograft survival in mice. Proc. Natl. Acad. Sci. USA.

[176] Lewis E.C., Mizrahi M., Toledano M., DeFelice N., Wright J.L., Churg A., Shapiro L., Dinarello C.A. (2008). Alpha1-antitrypsin monotherapy induces immune tolerance during islet allograft transplantation in mice. Proc. Natl. Acad. Sci. USA.

[177] Wang Y., Yan H.-J., Zhou S.-Y., Wang Y.-S., Qi H., Deng C.-Y., Li F.-R. (2014). The immunoregulation effect of alpha 1-antitrypsin prolong β-cell survival after transplantation. PLoS ONE.

[178] Lagarde W.H., Courtney K.L., Reiner B., Steinmann K., Tsalikian E., Willi S.M. (2021). Human plasma--derived alpha 1--proteinase inhibitor in patients with new--onset type 1 diabetes mellitus: A randomized, placebo--controlled proof--of--concept study. Pediatr. Diabetes.

[179] Rachmiel M., Strauss P., Dror N., Benzaquen H., Horesh O., Tov N., Weintrob N., Landau Z., Ben-Ami M., Haim A. (2016). Alpha-1 antitrypsin therapy is safe and well tolerated in children and adolescents with recent onset Type 1 diabetes mellitus. Pediatr. Diabetes.

[180] Brener A., Lebenthal Y., Interator H., Horesh O., Leshem A., Weintrob N., Loewenthal N., Shalitin S., Rachmiel M. (2018). Long-term safety of α-1 antitrypsin therapy in children and adolescents with Type 1 diabetes. Immunotherapy.

[181] Elshikha A.S., Lu Y., Chen M.-J., Akbar M., Zeumer L., Ritter A., Elghamry H., Mahdi M.A., Morel L., Song S. (2016). Alpha 1 antitrypsin inhibits dendritic cell activation and attenuates nephritis in a mouse model of lupus. PLoS ONE.

[182] Elshikha A.S., Yuan Y., Lu Y., Chen M.-J., Abboud G., Akbar M.A., Plate H., Wolney H., Hoffmann T., Tagari E. (2018). Alpha 1 antitrypsin gene therapy extends the lifespan of lupus-prone mice. Mol. Ther. Methods Clin. Dev..

[183] Singh B.B., Ohm J., Quenum Zanbede F.O., Chauhan P., Kroese F.G.M., Vissink A., Ambrus J.L., Mishra B.B. (2020). Decrease in alpha-1 antiproteinase antitrypsin is observed in primary Sjogren’s syndrome condition. Autoimmunity.

[184] Marcondes A.M., Hockenbery D., Lesnikova M., Dinarello C.A., Woolfrey A., Gernsheimer T., Loghman-Adham M., Gelmont D., Storer B., Hansen J.A. (2016). Response of steroid-refractory acute GVHD to α1-antitrypsin. Biol. Blood Marrow Transplant..

[185] Magenau J.M., Goldstein S.C., Peltier D., Soiffer R.J., Braun T., Pawarode A., Riwes M.M., Kennel M., Antin J.H., Cutler C.S. (2018). α1-antitrypsin infusion for treatment of steroid-resistant acute graft-versus-host disease. Blood.

[186] Strassmair M., Stangl M. (2021). Alpha-1 antitrypsin deficiency and COVID-19 infection. Arch. Bronconeumol..

[187] McEvoy N.L., Clarke J.L., Mc Elvaney O.J., Mc Elvaney O.F., Boland F., Hyland D., Geoghegan P., Donnelly K., Friel O., Cullen A. (2021). A randomised, double-blind, placebo-controlled, pilot trial of intravenous plasma purified alpha-1 antitrypsin for SARS-CoV-2-induced Acute Respiratory Distress Syndrome: A structured summary of a study protocol for a randomised, controlled trial. Trials.

[188] McElvaney O.J., O’Connor E., McEvoy N.L., Fraughan D.D., Clarke J., McElvaney O.F., Gunaratnam C., O’Rourke J., Curley G.F., McElvaney N.G. (2021). Alpha-1 antitrypsin for cystic fibrosis complicated by severe cytokinemic COVID-19. J. Cyst. Fibros..

[189] Ritzmann F., Chitirala P., Krüger N., Hoffmann M., Zuo W., Lammert F., Smola S., Tov N., Alagem N., Lepper P.M. (2021). Therapeutic application of alpha-1-antitrypsin in COVID-19. Am. J. Respir. Crit. Care Med..

